# Design and Synthesis of Novel Hybrid Molecules against Malaria

**DOI:** 10.1155/2015/458319

**Published:** 2015-02-05

**Authors:** Melanie Lödige, Luisa Hiersch

**Affiliations:** Institute of Organic Chemistry, University of Würzburg, Am Hubland, 97074 Würzburg, Germany

## Abstract

The effective treatment of malaria can be very complex: *Plasmodium* parasites develop in multiple stages within a complex life cycle between mosquitoes as vectors and vertebrates as hosts. For the full and effective elimination of parasites, an effective drug should be active against the earliest stages of the *Plasmodium* infection: liver stages (reduce the progress of the infection), blood stages (cure the clinical symptoms), and gametocytes (inhibit the transmission cycle). Towards this goal, here we report the design, the synthetic methodology, and the characterization of novel hybrid agents with combined activity against *Plasmodium* liver stages and blood stages and gametocytes. The divergent synthetic approach allows the access to differently linked primaquine-chloroquine hybrid templates in up to eight steps.

## 1. Introduction

Malaria is still one of the most menacing infectious diseases worldwide with estimated 207 million clinical cases and 627,000 death cases (WHO; [1]). The treatment of malaria is complicated by the increasing development of resistance to the currently used medicaments and by the pathogen's unique biological characteristics [2]. The intracellular and unicellular* Plasmodium* parasites [3] develop within a complex life cycle between mosquitoes as vectors and vertebrates as hosts [4].* Plasmodium* sporozoites are transmitted through the bite of anopheles mosquitoes to humans [5, 6] and are transported by the bloodstream to the liver cells. Therein, they develop into preerythrocytic forms [6] and finally transform into erythrocytes infecting [4, 7] merozoites [8]. These merozoites mature into schizonts, followed by the rupture of red corpuscles and the release of thousands of merozoites that reinfect erythrocytes and in the end continue the circle of asexual reproduction [9]. A few merozoites develop into gametocytes that are transmitted back to the female anopheles mosquitoes during the blood meal [10], finally completing the malaria transmission cycle [6]. All clinical symptoms such as fever, anemia, splenomegaly [9], and neurological impairment are associated with the parasitic blood stages [7], and the severity of symptoms depends on the patients' immune status [9].

Among the human pathogenic malarial parasites,* Plasmodium falciparum* is the most dangerous one [11], responsible for more than 90% of all death cases [12]. Unlike* P. falciparum*,* Plasmodium vivax* and* Plasmodium ovale* have a particular characteristic: they produce hypnozoites [11, 13]. The hypnozoites remain quiescent in the liver cells [14] for several weeks up to several years until their activation causes a relapse without a new infectious bite [6].* Plasmodium* parasites have another peculiar characteristic: their surface antigens are different in many parts in each developmental stage. With this complex life cycle, in each stage the parasites can be considered as a different organism, although all stages are attributed to the same genome [15]. This complicates the treatment of malaria that should be active against as many stages as possible; thus finding new effective compounds will help current therapies.

Taking this into account, we have reported here the design, synthesis, and characterization of novel hybrid molecules consisting of the well-known antimalarial drugs primaquine (**1**) and chloroquine (**2**). The most active hybrid compounds of our study are potential new drug templates for the treatment of malaria and show activities against the* Plasmodium* liver stages and blood stages and against gametocytes [16, 17]. The synthetic methodology uses a divergent synthetic approach to differently linked primaquine-chloroquine hybrid templates, resulting in several molecules based on one key intermediate (**12**).

The new hybrids reported in this work showed good to excellent biological activity against the liver stages (*P*.* berghei*), blood stages (*P*.* falciparum*, strains 3D7, Dd2, and K1), and gametocytes (*P*.* falciparum*); the activity is better than the activity of the parent drugs primaquine (**1**) and chloroquine (**2**) combined. The detailed biological results are part of a more comprehensive study described and published in our patent application [16, 17].

## 2. Results and Discussion

### 2.1. Basic Considerations for the Design of Hybrid Molecules against Malaria

Sporozoites and liver stages are fascinating targets for the development of new drugs. Effective drug molecules against the earliest stages would decrease or even avoid the occurrence of blood stages and, consequently, of clinical symptoms (prophylactic effectiveness) [14]. In addition, active compounds against gametocytes would fully eradicate the parasites, thereby efficiently preventing the transmission cycle [14]. However, more than 90% of the current drug research projects worldwide aim at targeting the blood stages [6]. At present, there is no medicament available equally active against all stages of the life cycle and against all* Plasmodium* species [18]. In order to improve the malaria treatment, the World Health Organization recommended in 2001 to combine antimalarial drugs and to avoid the monotherapeutic treatment [19]. Combination therapy may overcome pharmacodynamic disadvantages [20], but resistance may develop when the drugs have different half-life values or have in general long half-lives, thus reaching subtherapeutic blood concentrations [21–24]. Additionally, patients often do not comply with complex treatment schemes of multiple medicaments [25].

The hybrid concept gained importance in the last decade. The synthesis of hybrid molecules of two or more [26] established drugs (full or partial molecules) and of natural product structures was used more and more and resulted in synergistic effectiveness, especially against resistant organisms by these new structures [27]. A hybrid drug has a single pharmacokinetic profile, easy to predict and control [28, 29], thus superior to a standard combination therapy [30]; hybrid drugs are absorbed, distributed, metabolized, and excreted at one single rate [20]. With hybrid drugs there is no competition for plasma protein binding as in the case of single drugs [20], thus reducing the risk of drug interactions [31]. The ratio of the single drugs is determined by the hybrid structure, and doses cannot be as flexibly administered as in the case of single drugs [32]. In addition, pharmacokinetic characteristics can be controlled by the linkage moiety [33]. For a successful hybrid drug, the activities of each single drug should be within the same concentration range to prevent the activity of only one moiety [20]. In conclusion, hybrid molecules can offer the advantages of a combination therapy along with improved pharmacokinetic profiles and potential activity against resistant strains by these new structures but have the disadvantage of a less flexible administration.

The use of established drugs can result in a rapid therapeutic progress with low economical risk [34, 35]. To avoid the vicious circle of a* Plasmodium* infection, an ideal drug would have good activity against sporozoites (prophylactic activity) [36], preerythrocytic liver stages (causal-prophylactic) [37, 38], blood stages (suppressive-prophylactic and therapeutic activity), and gametocytes [39, 40] as well as against hypnozoites to avoid relapses [41].

The drugs primaquine (**1**) and chloroquine (**2**) have been successfully used in combination for the weekly chemoprophylaxis against malaria during the Vietnam War [42–44]. Both are approved and low-priced drugs [45], and they are active against most stages of the* Plasmodium* infection in humans. Primaquine (**1**) has a very short half-life [41, 46] and accumulates potentially harmful metabolites [47, 48]. However, primaquine has been safe and an effective prophylactic agent for nonpregnant women and travelers with a normal glucose-6-phosphate dehydrogenase level [42]: in endemic malaria regions up to 20% of the patients on average suffer from such a deficiency [49]. Chloroquine has a long terminal half-life with a higher risk to develop resistant strains under subtherapeutic concentrations [50, 51] and is a safe medicament in therapeutic doses [52]. In addition, primaquine can reduce the chloroquine efflux transport (a resistance mechanism) by binding to the PfCRT channels [53] and support the activity of chloroquine [54].

The goal of our study was to design and synthesize compounds active against different* Plasmodium* infection stages by the combination of structural motives of both compounds in one hybrid molecule.

### 2.2. Chemistry

**Figure 1:**
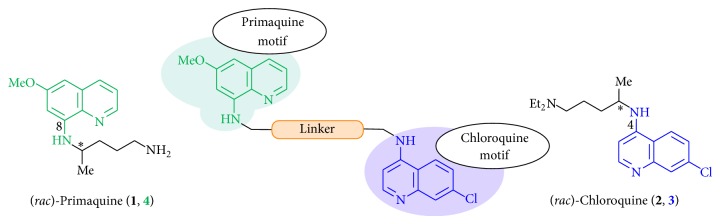
The first component of the hybrid drugs (*rac*)-primaquine (**1**) and its pharmacophore moiety (in green,** 4**) as well as the second drug (*rac*)-chloroquine (**2**) and the corresponding pharmacophore structure (in blue,** 3**).

**Figure 2:**
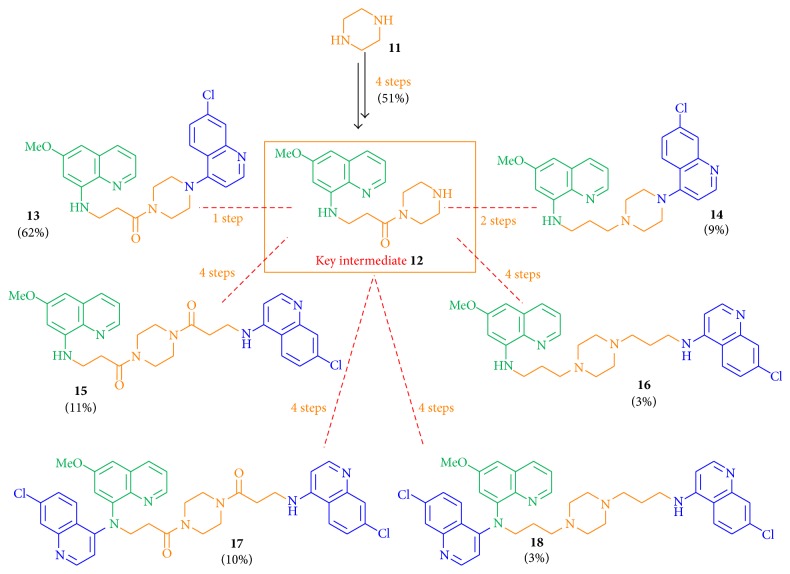
Overall view of the piperazine-linked hybrid compounds** 13** to** 18**, which were synthesized by the divergent synthetic route starting from piperazine (**11**).

Hybrid molecules of primaquine (**1**) and chloroquine (**2**) pharmacophores were designed and synthesized with different types of linkage: with an authentic linker part, without a linkage moiety, with an elongated piperazine diamide or diamine linker bond, and with an aromatic-type linkage (Figures 1 and 2) [16, 17].

#### 2.2.1. Primaquine-Chloroquine Hybrid Molecules with Authentic Linkage

**Scheme 1:**
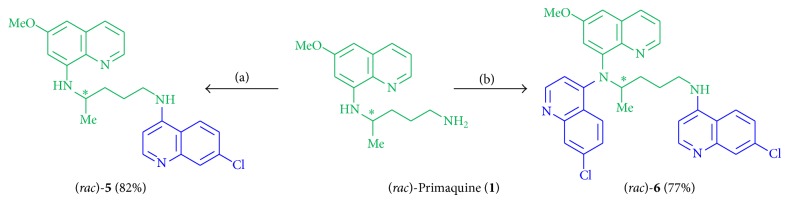
Synthesis of the primaquine-chloroquine hybrid molecules (ratio 1 : 1 [57] and ratio 1 : 2) with an authentic linkage part of primaquine (**1**). Reagents and conditions: (a) 4,7-dichloroquinoline (0.5 equivalents), neat, 120°C; (b) 4,7-dichloroquinoline (3 equivalents), neat, 120°C.

The first dual molecules were synthesized with an authentic linkage part in order to avoid additional structural moieties on the pharmacodynamic and pharmacokinetic properties of the hybrid compounds. Earlier examples of primaquine derivatives showed that the linkage of primaquine can be performed via the primary amine without loss of activity [55, 56]. Therefore, hybrid structures of primaquine (**1**) and a chloroquine moiety (**3**) were synthesized with a linker using original side chain of primaquine, with a pharmacophore ratio of 1 : 1 and 1 : 2 of primaquine (**1**) to chloroquine (**2**). Since a Buchwald-Hartwig amination protocol leads merely to low yields (a decomposition of the palladium catalyst was observed), a nucleophilic substitution reaction of primaquine (**1**) and 4,7-dichloroquinoline was performed at 120°C under neat conditions to obtain the hybrid molecules (*rac*)-**5** (82% yield) and (*rac*)-**6** (77%, Scheme 1) [57].

#### 2.2.2. Primaquine-Chloroquine Hybrid Molecules without Linkage

Additional derivatives consisting of the aromatic pharmacophore were prepared by a direct linkage of both motives in a ratio of 1 : 1 and 1 : 2 of primaquine (**4**) to chloroquine (**3**) moiety.

**Scheme 2:**
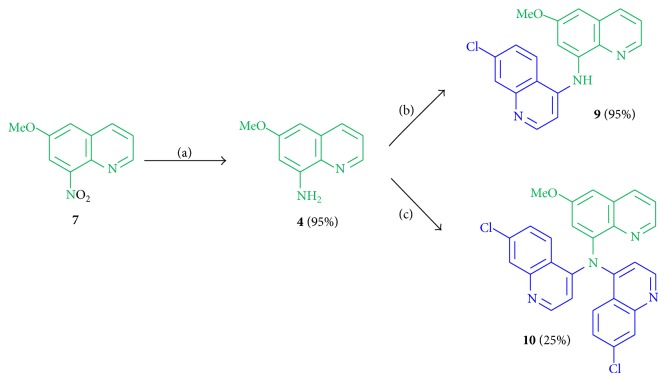
Synthesis of the hybrid compounds** 9** and** 10**. Reagents and conditions: (a) H_2_ (g), Pd/C, MeOH (dry), 25°C; (b) 4,7-dichloroquinoline (0.7 equivalents), neat, 120°C; (c) 4,7-dichloroquinoline (3 equivalents), neat, 120°C.

The commercially available 6-methoxy-8-nitroquinoline (**7**) was hydrogenated by hydrogen gas and Pd/C as catalyst in MeOH (dry) to give the amine** 4** in 95% yield. This primaquine motif was allowed to react with 4,7-dichloroquinoline at 120°C under neat conditions to give the hybrid molecules** 9** in 95% and** 10** in 25% yield (Scheme 2).

#### 2.2.3. Divergent Synthetic Route to Piperazine-Linked Primaquine-Chloroquine Hybrid Molecules

The diamine piperazine (**11**) can link the pharmacophore moieties** 3** and** 4** in different ways: through one or both nitrogen atoms and through amide or amine bonds. By using a divergent synthetic route, six hybrid molecules (**13** to** 18**) were obtained starting from the same educt piperazine (**11**, Figure 2) in up to eight steps. The synthesized hybrid molecules differ in their number of nitrogen atoms and in their type of bond (amide or amine bond) that determines their basic molecular properties. The dual molecules consist at least of two quinoline nitrogen atoms, of two anilinic nitrogen atoms, and of either one or two basic tertiary piperazine amine functionalities (**14**,** 16**, and** 18**) or of one and two nonbasic tertiary piperazine amide functions (**13**,** 15**, and** 17**). Thus, the compounds differ in the possibility to be enriched by protonation in acidic compartments (described for chloroquine [58]), thus influencing the bioactivity against the parasitic stages.

**Scheme 3:**
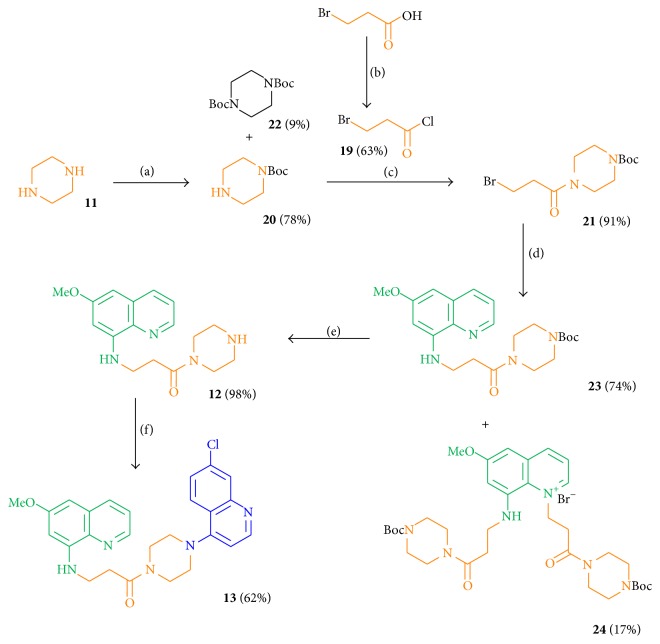
Synthetic route for hybrid molecule** 13**, a piperazine-linked primaquine-chloroquine dual molecule. Reagents and conditions: (a) Boc_2_O, DCM (dry), 0°C; (b) SOCl_2_, 80°C; (c) NaOAc, DCM (dry), 0–25°C; (d) 6-methoxy-8-aminoquinoline (**4**), NaH, DMF (dry), 0–25°C; (e) TFA, DCM, 25°C; (f) 4,7-dichloroquinoline, neat, 120°C.

All piperazine-linked hybrid molecules contained a C3 spacer between the primaquine pharmacophore and the piperazine part. The required acyl chloride** 19** for the spacer part was produced in 63% yield by the reflux of 3-bromopropionic acid with thionyl chloride [59]. The sequence started with piperazine, which was first protected by a Boc group using Boc_2_O in DCM (dry) at 0°C to obtain the monoprotected piperazine** 20** in 78% yield. Compound** 20** was further converted into the bromine derivative** 21** in 91% yield by slowly dropping** 19** into a solution of** 20** in DCM (dry) at temperatures from 0°C to 25°C (Scheme 3) [60].

6-Methoxy-8-aminoquinoline (**4**) was deprotonated using NaH in DMF (dry) at 0°C and was allowed to react with the bromine-substituted linker part** 21** at temperatures from 0°C up to 25°C to obtain compound** 23** in good yields (74%) and the disubstituted side product** 24** in 17% yield. Performing the Boc deprotection of amine** 23** using TFA, the free amine and key intermediate** 12** of the divergent route was obtained in 98% yield and was linked to 4,7-dichloroquinoline at 120°C under neat conditions to give hybrid** 13** in 62% (Scheme 3).

**Scheme 4:**
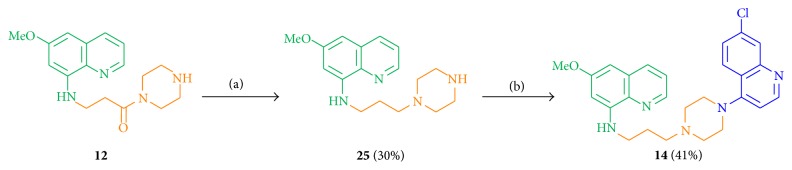
Synthesis of the hybrid substance** 14**, starting with the reduction of the key intermediate** 12** and followed by the linkage to the chloroquine motif. Reagents and conditions: (a) LiAlH_4_, THF (dry), 80°C; (b) 4,7-dichloroquinoline, neat, 120°C.

The key intermediate** 12** was reduced by LiAlH_4_ in THF (dry) to obtain compound** 25** in low yields of 30% (Scheme 4). Multiple reasons for the low reduction yields of primaquine analogs have been described earlier [61]: formation of numerous soluble and stable complexes of the primaquine moiety with the reducing agent LiAlH_4_ at room temperature [61], which complicates the detection of the complete consumption of the starting material during the reaction. Furthermore, a splitting of the 8-amine-carbon bond by reducing agent LiAlH_4_ and a partial reduction of the pyridinic moiety to the corresponding tetrahydroisoquinoline might have caused low yields [61]. Conversion of** 25** with 4,7-dichloroquinoline at 120°C under neat conditions gave compound** 14** in 41% yield.

**Scheme 5:**
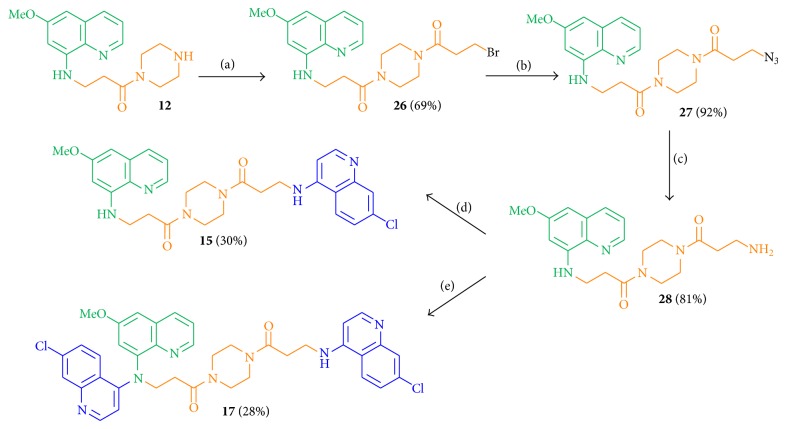
Synthesis of the hybrid compound** 15** (ratio 1 : 1) and of substance** 17** (ratio 1 : 2) by the divergent synthetic route using a C3-piperazine-C3 linkage. Reagents and conditions: (a) 3-bromopropionyl chloride (**19**), NaOAc, DCM (dry), −20°C; (b) NaN_3_, DMF (dry), 25°C; (c) PPh_3_, MeOH (dry), 25°C; (d) 4,7-dichloroquinoline (0.75 equivalents), neat, 120°C; (e) 4,7-dichloroquinoline (3 equivalents), neat, 120°C.

The synthesis of hybrid molecules with a further elongated C3-piperazine-C3 linkage started with the introduction of the additional C3 part to the key intermediate** 12**, which was allowed to react with the acyl chloride** 19** at −20°C to give amide** 26** in 69% yield (Scheme 5), and the two additional regioisomers as well as the corresponding elimination product of amide** 26**. The azide function was introduced to** 26** using NaN_3_ to obtain the azide** 27** in 92% yield, followed by the reduction to the amine** 28** in 81% yield using the Staudinger reaction. The linkage of amine** 28** with 4,7-dichloroquinoline gave the dual molecule** 15** or the disubstituted hybrid** 17** (depending on the number of equivalents of 4,7-dichloroquinoline) in low yields of 30% (**15**) and 28% (**17**), respectively.

**Scheme 6:**
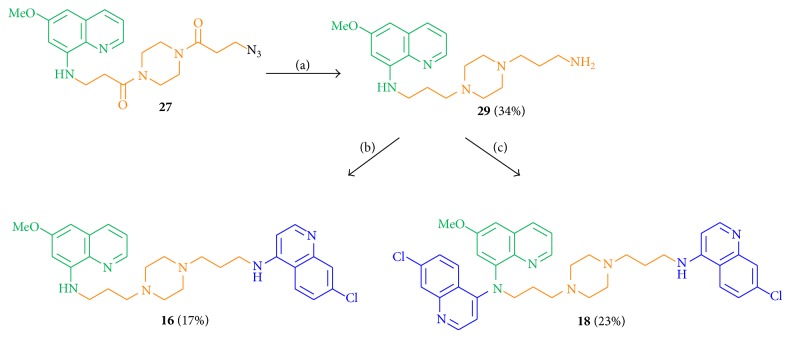
Synthetic route of the hybrid molecules** 16** and** 18** with higher basic molecule properties. Reagents and conditions: (a) LiAlH_4_, THF (dry), 80°C; (b) 4,7-dichloroquinoline (0.8 equivalents), neat, 120°C; (c) 4,7-dichloroquinoline (five equivalents), neat, 120°C.

Starting from the azide** 27**, two further hybrid molecules (**16**,** 18**) were obtained. Azide** 27** was reduced using LiAlH_4_ in 34% yield to amine** 29** with two tertiary and basic piperazine nitrogen atoms that might influence the bioactivity values by enrichment in acidic compartments. The free amine function of compound** 29** was linked with 4,7-dichloroquinoline to the monosubstituted** 16** and the disubstituted** 18** in 17% and 23% yield, respectively (Scheme 6).

#### 2.2.4. Synthetic Route to Hybrid Molecule 30 with an Aromatic-Type Linkage

The influence of the linkage composition on the bioactivity values was further investigated, and a hybrid molecule with a nonbasic, plain aromatic linker moiety was designed and synthesized. The distance between the linkage positions of the primaquine pharmacophore and of the chloroquine pharmacophore moieties corresponded approximately to the distance in the genuine hybrid molecule** 5**. The number of nitrogen atoms was not changed, but the lipophilicity was expected to be higher than that for the genuine compound** 5** due to the formal* para*-xylene part.

The first attempts to synthesize** 30** from the less expensive chloroquine moiety were not successful. Therefore, the synthesis was started from the primaquine building block.

**Scheme 7:**
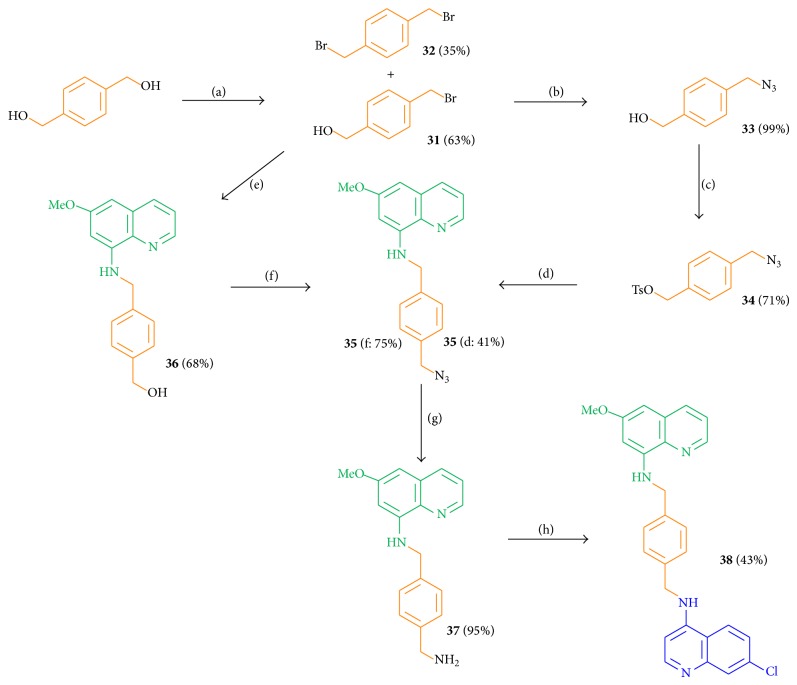
Two variants of the protecting group free synthesis of the aromatic-type linker moiety to obtain the key intermediate azide** 35**. Reagents and conditions: (a) (CBrCl_2_)_2_, PPh_3_, DCM (dry), 0–25°C; (b) NaN_3_, DMF (dry), 25°C; (c) TsCl, NaH, DCM (dry), 0–25°C; (d) 6-methoxy-8-aminoquinoline (**4**), NaOAc, DMF (dry), 25°C; (e) 6-methoxy-8-aminoquinoline (**4**), NaOAc, DMF (dry), 25°C; (f) DPPA, DBU, toluene (dry), 25°C; (g) PPh_3_, MeOH (dry), 25°C; (h) 4,7-dichloroquinoline, neat, 120°C.

The protecting group free protocol started with the commercially available 1,4-benzenedimethanol, which was converted by the use of 1,2-dibromotetrachloroethane and PPh_3_ to the monobrominated product** 31** in 63% yield and to the side product, the double brominated compound** 32**, in 35% yield (Scheme 7). The introduction of the azide function by NaN_3_ gave 99% of compound** 33**, followed by the tosylation with* p*-toluenesulfonyl chloride to compound** 34** in 71% yield and by the linkage to the primaquine motif** 4** yielding compound** 35** (41%). The key intermediate** 35** was also obtained by the linkage of the brominated derivative** 31** with 6-methoxy-8-aminoquinoline (**4**) in 68% yield, followed by the azide introduction using DPPA and DBU in toluene (dry) at room temperature in 75% yield [62]. The first route over four steps resulted in the key intermediate** 35** with an overall yield of 18%; the second route over three steps resulted in intermediate** 35** with a yield of 32%.

Compound** 35** was reduced to amine** 37** (95%) by Staudinger conditions; the amine** 37** was finally linked to the second pharmacophore moiety using 4,7-dichloroquinoline at 120°C under neat conditions, yielding the target hybrid molecule** 38** in 43%.

### 2.3. Discussion

**Scheme 8:**
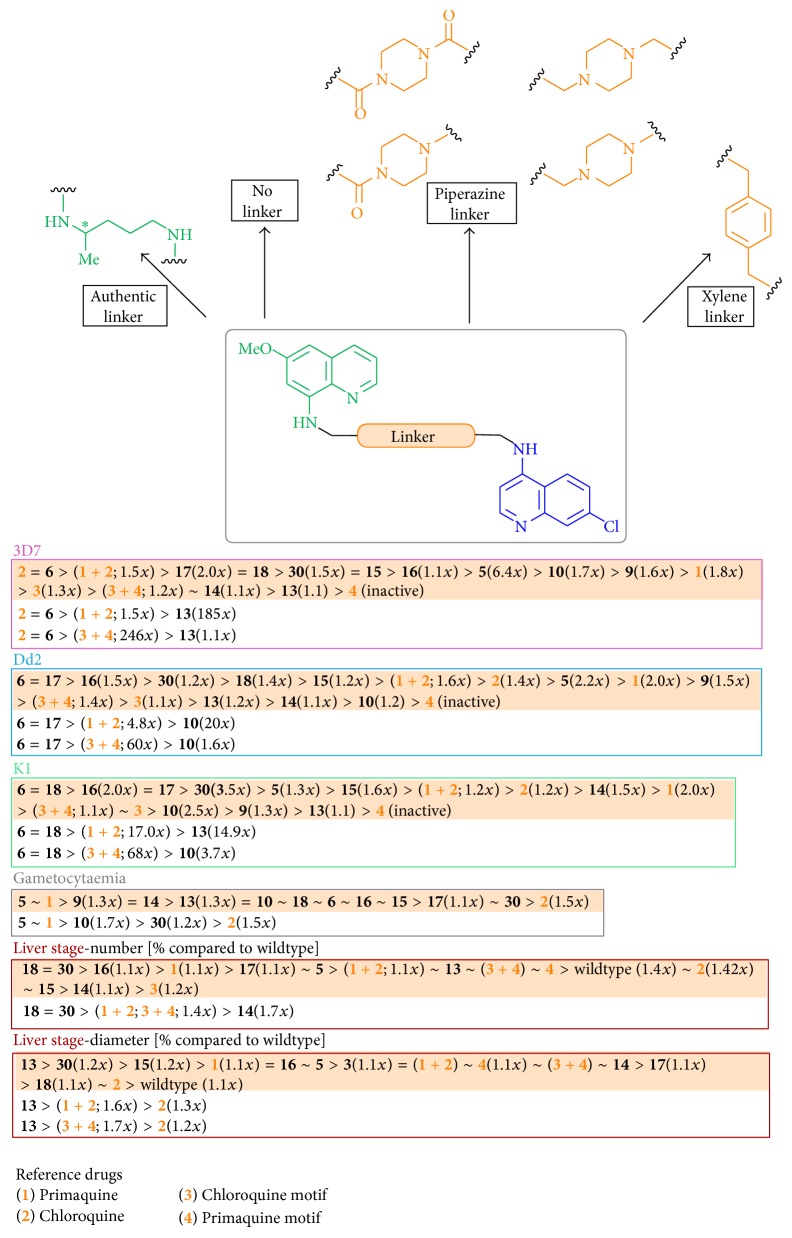
Bioactive molecules of the study, reference drugs (**1**,** 2**,** 3**, and** 4**), and their combinations (1 + 2, 3 + 4) in order from the highest to the lowest activities (referring to IC_50_ values for 3D7, Dd2, and K1, gametocytaemia, and number and diameter of the liver stages, resp.); in brackets the ratios of activities of the compounds compared to the former substances.

Hybrid** 5** showed a significant effect on the morphology of the liver and on the total number of liver stage parasites (*P*.* berghei*), which were reduced by a concentration significantly lower than that of primaquine (**1**) required for the same activity. All additional hybrid molecules showed an influence on the size of liver stages in comparison with the parasite wildtype and on the number of liver stages after 24 hours (Scheme 8).

Amide** 13** compared with amine** 14** showed a decreased number of liver stages and a greater influence on the growth with a smaller diameter of the parasitic stages. The number of liver stages and the parasite growth were slightly decreased using the elongated amide** 15** compared with the** 14** with a second C3 linker; in comparison with** 15**, the amine** 16** showed a stronger increase in the number of parasites, whereas the diameter did not alter significantly. The introduction of the second elongating linker part as well as the variation of the basic molecule properties of the four investigated molecules (**13** to** 16**) had very little influence on the activity against the number and the diameter of parasites. The introduction of two chloroquine motives resulted in molecules that were less effective against the growth compared with the previous monosubstituted derivatives: amide** 17** and the corresponding amine** 18** showed a comparable weak effect on the growth of parasites, but after 24 hours** 17** and** 18**, as well as** 16** and** 38**, showed the lowest number of liver stages. In this case as well, a modification of the basic molecule property values did not influence the activity against liver stages.

The hybrid molecules were tested against the following strains of* P. falciparum*: 3D7 (chloroquine and pyrimethamine sensitive strain) [63, 64], Dd2 (resistant against chloroquine, mefloquine, and pyrimethamine) [65], and K1 (resistant against pyrimethamine [63, 66] and one of the most resistant strains against chloroquine) [67]. The equimolar combination of primaquine (**1**) and chloroquine (**2**) and the equimolar combination of both the chloroquine (**3**) and the primaquine motives (**4**) were measured in comparison with the hybrid compounds.

Almost all of the investigated hybrid molecules showed improved activity against blood stages of* P*.* falciparum* (3D7, Dd2, and K1). Against strain 3D7, hybrid molecule** 5** showed lower activity than chloroquine, but** 5** was still very active at a concentration lower than 1 *µ*M, also in comparison with primaquine and to the chloroquine motif** 3**. The primaquine moiety** 4** was not active. The combination of both the chloroquine and primaquine motifs** 3** and** 4** showed activity in the range of the chloroquine motif alone and hence had no additional or synergistic effect on each other. Also, the equimolar combination of primaquine (**1**) and chloroquine (**2**) showed activity in the concentration range of chloroquine.

Against strain Dd2, hybrid** 5** showed a slightly improved activity compared with 3D7. Chloroquine (**2**) was less active against Dd2 than against 3D7, and primaquine (**1**) had only a weak effect. The primaquine motif** 4** showed no activity, but the chloroquine motif** 3** surprisingly showed a better activity compared with 3D7. This was also confirmed by the equimolar combination of both motives** 3** and** 4**. The equimolar combination of chloroquine (**2**) and primaquine (**1**) showed enhanced activity compared with only chloroquine (**2**), thus hinting the chloroquine resistance-reversing effect of primaquine (**1**) [54].

Against strain K1, the equimolar combination of chloroquine (**2**) and primaquine (**1**) showed a better activity than against the strain Dd2. Chloroquine (**2**) showed a comparable activity in comparison with Dd2, and the order of efficacy of primaquine (**1**) was 3D7 < Dd2 < K1. Also, the hybrid compound** 5** showed the best activity against the K1 strain. The order of activity of compound** 5** was 3D7 ≈ Dd2 < K1 (five times higher than Dd2 and 3D7). This higher activity against K1 strain than against the other strains can be probably due to the resistance-reversing effect of primaquine [54], combined with the better activity of primaquine on its own. The primaquine moiety** 4** was inactive against K1, but surprisingly the chloroquine motif** 3** showed its best activity against K1. The combination of both motifs** 3** and** 4** showed effectiveness against K1 in the same range as** 3** against K1 due to presence of the chloroquine motif.

Both compounds** 9** and** 10** showed good activities against all strains but were slightly less active than the first representative** 5**. The derivative** 6** showed improved bioactivities in the submicromolar range and had a similar linkage as compound** 5** originating from the primaquine side chain but with a pharmacophore ratio of primaquine to chloroquine moiety of 1 : 2. This might be due to not only the number of pharmacophores but also an increased lipophilicity correlating with an enhanced membrane permeability, an increased basicity with a higher enrichment in acidic compartments, or an increased planar, aromatic system capable of shielding the surface of hemozoin from further growth.

The piperazine-linked amide** 13** and the amine** 14** showed the lowest activities against the blood stages. Only** 14** showed very good activity against strain K1 (lower than 1 *µ*M). The low activity of both these molecules could be due to the lack of a strong basic center such as the tertiary nitrogen atom, like in compound** 16**.

Compounds** 15** and** 16**, elongated piperazine derivatives with a second C3 linker, showed improved activities against 3D7 and Dd2 whereby the diamine** 16**, with higher basic properties, was significantly active against strain K1. This might be influenced by a higher total flexibility of compounds** 15** and** 16** due to two C3 linkers; the basic properties of the diamide** 15** and the diamine** 16** might not be the cause of their higher activity, since they are both significantly more effective against all strains compared with hybrid** 5**. A higher ratio of primaquine to chloroquine motifs of 1 : 2 resulted in a slightly higher activity for** 17** and** 18** compared with the very good activities of the derivatives** 15** and** 16**.

A combination of linkage properties and the pharmacophore ratio, such as for compound** 6**, seemed to influence the activity more than just the ratio of primaquine and chloroquine by itself (compounds** 15**,** 16** compared to** 17**,** 18**). Also compound** 30** with an aromatic-type moiety as linkage and a comparable distance for the pharmacophore moieties as in compound** 5** but with higher lipophilicity properties and a possible higher hemozoin shielding (**30**) showed very good activities, lower than 0.1 *µ*M against all strains.

In conclusion, hybrid molecules** 5**,** 6**,** 15**–**18**, and** 30** all showed excellent activity values against the tested strains 3D7, Dd2, and K1 of* P. falciparum* with concentrations lower than 0.1 *µ*M: ten times higher than the definition of a* hit substance* required in drug development [68]. These compounds also showed activity in the concentration range of equimolar combination of substances primaquine (**1**) and chloroquine (**2**) or even better.

The inhibition of the development of blood stages to gametocytes and the death of gametocytes are important to block the vicious circle of transmission, reinfection, and spread of resistant organisms [69, 70]. Although a gametocidal activity is required for a successful hybrid molecule, the compounds that are active against blood stages should not induce the formation of gametocytes [71], which can be generally induced by medicaments in subtherapeutic concentrations, since the parasite is able to vary the number of gametocytes to ensure the survival [72, 73].

The influence of primaquine (**1**) on gametocytaemia was used as positive control. As expected, chloroquine (**2**) showed a neglectable influence. The hybrid molecules all influenced the development of gametocytes. The best activity was obtained for the linker-free compounds** 9** and** 10**, the C3-linked piperazine derivative** 14**, and the first representative** 5**. Compound** 6**, the amide** 13**, the diamide** 15**, the diamine** 16**, the diamide** 17**, the diamine** 18**, and compound** 30** were all comparably active. Interestingly, the linkage moiety, the basicity, and the pharmacophore ratio seemed to barely influence the activity against gametocytes.

### 2.4. Conclusion

We presented in summary eleven hybrid molecules of primaquine (**1**) and chloroquine (**2**) synthesized in eight or less synthetic steps, investigated for their activity against the* Plasmodium* liver stages, blood stages, and gametocytes (Scheme 8). In conclusion, we showed that it is possible to design and synthesize hybrid drug molecules that have good to excellent bioactivity values against different stages of the* Plasmodium* infection. We also analyzed the influence on the development of liver stages in hepatocytes (*P. berghei*), the activity against blood stages of three different strains (3D7, Dd2, and K1 of* P. falciparum*), and their efficacy against the maturation of gametocytes (*P. falciparum*) [16, 17]. In general, it is important to highlight that hybrid molecules often do not follow structural activity relationship rules of the single components. The most promising structure of the synthesized compounds was the hybrid molecule** 30** with the highest activity against the number and the diameter of liver stages, against blood stages of 3D7, Dd2, and K1 (lower than 0.1 *µ*M), and against gametocytes. This novel class of hybrid molecules shows activity against all stages of the* Plasmodium* infection in humans and also against resistant strains [16, 17].

## 3. Experimental Section

### 3.1. General Information

All solvents were distilled before use. Commercially available materials were used without further purification, purchased by Sigma-Aldrich. Thin layer chromatography was carried out using silica gel 60 *F*_254_ or alumina with fluorescent indicator. The compounds were detected by fluorescence quenching at 254 nm, fluorescence at 356 nm, or staining with iodine or ninhydrin. Flash chromatography was performed using silica gel (20–63 mesh), deactivated silica gel (20–63 mesh; 7.5% ammonia), or ICN neutral or basic alumina, deactivated with 15% H_2_O. NMR spectra were obtained on a Bruker DMX 600 apparatus and are reported in ppm relative to internal solvent signal with coupling constants (*J*) in Hertz (Hz). Spectra were usually obtained at 25°C. EI mass spectrometry was carried out on a Finnigan MAT 8200; ESI-HRMS was measured on a Bruker Daltonik micrOTOF-focus.

### 3.2. Synthesis and Characterization of the Hybrid Molecules

#### 3.2.1. *N* ^1^,*N* ^4^-Bis(7-chloroquinolin-4-yl)-*N* ^4^-(6′-methoxyquinolin-8′′-yl)pentane-1,4-diamine (**6**)

A total of 197.9 mg (0.76 mmol) of the free base of primaquine (**1**) and 477.6 mg (2.41 mmol) of 4,7-dichloroquinoline were heated at 120°C under neat conditions for 7.5 hours. The reaction mixture was allowed to cool down, followed by the suspension of the residue in a solvent mixture of dichloromethane and small amounts of methanol and by the addition of some drops of ammonia solution to alkalize the mixture. The solvent mixture was removed under reduced pressure; the residue was suspended in dichloromethane, filtered (Celite), and concentrated.

Purification with flash column chromatography on deactivated silica gel (gradient elution starting with petroleum ether/ethyl acetate 1 : 1 and followed by 1 : 2) yielded compound** 6** (340.7 mg, 77%) as yellow crystals. Mp 109°C (petroleum ether/ethyl acetate), IR (ATR-FTIR): $\tilde{v}$ = 3627–3000 (w, br), 2935 (w), 2854 (w), 2515 (w), 2448 (w), 2360 (s), 2341 (s), 1698 (w), 1637 (w), 1604 (m), 1573 (s), 1503 (m), 1446 (m), 1408 (m), 1372 (m), 1292 (w), 1232 (w), 1211 (w), 1157 (w), 1137 (w), 1107 (w), 1074 (w), 1021 (w), 980 (w), 916 (w), 877 (w), 845 (w), 808 (m), 783 (m), 680 (w), 669 (w), 644 (w), 626 (w), 617 (w) cm^−1^. ^1^H-NMR (600 MHz, MeOD-d_4_): *δ* = 1.40 (t, ^3^ *J*_H–H_ = 5.61 Hz, 6 H, Me), 1.87–2.01 (m, 8 H, 2-CH_2_, 3-CH_2_), 3.40–3.44 (m, 4 H, 1-CH_2_), 3.65 (s, 3 H, OMe), 3.66 (s, 3 H, OMe), 3.93–3.96 (m, 2 H, 4-H), 6.42 (d, ^3^ *J*_H–H_ = 5.70 Hz, 1 H, 3′′′-H), 6.44 (d, ^3^ *J*_H–H_ = 5.64 Hz, 1 H, 3′′′-H), 6.63 (s, 1 H, 7′-H), 6.65 (s, 1 H, 7′-H), 7.15–7.19 (m, 2 H, 3′-H), 7.27–7.39 (m, 12 H, 4′-H, 5′-H, 3′′-H, 5′′-H, 6′′-H, 6′′′-H), 7.72 (d, ^4^ *J*_H–H_ = 2.10 Hz, 1 H, 8′′′-H), 7.73 (d, ^4^ *J*_H–H_ = 2.10 Hz, 1 H, 8′′′-H), 8.00 (d, ^3^ *J*_H–H_ = 8.94 Hz, 1 H, 5′′′-H), 8.04 (d, ^3^ *J*_H–H_ = 9.00 Hz, 1 H, 5′′′-H), 8.07 (t, ^4^ *J*_H–H_ = 1.50 Hz, 2 H, 8′′-H), 8.18 (d, ^3^ *J*_H–H_ = 5.70 Hz, 1 H, 2′′′-H), 8.19 (d, ^3^ *J*_H–H_ = 5.64 Hz, 1 H, 2′′′-H), 8.46 (dd, ^3^ *J*_H–H_ = 4.08 Hz, ^4^ *J*_H–H_ = 1.50 Hz, 1 H, 2′-H), 8.47 (dd, ^3^ *J*_H–H_ = 4.08 Hz, ^4^ *J*_H–H_ = 1.50 Hz, 1 H, 2′-H), 8.89 (d, ^3^ *J*_H–H_ = 4.56 Hz, 1 H, 2′′-H), 8.90 (d, ^3^ *J*_H–H_ = 4.56 Hz, 1 H, 2′′-H) ppm. ^13^C-NMR (150 MHz, MeOD-d_4_): *δ* = 21.15 (Me), 21.18 (Me), 25.85 (C-2), 26.18 (C-2), 35.09 (C-3), 35.35 (C-3), 44.01 (C-1), 44.08 (C-1), 49.08 (C-4), 49.21 (C-4), 56.64 (OMe), 93.00 (C-7′), 93.05 (C-7′), 99.78 (C-3′′′), 99.79 (C-3′′′), 105.48 (C-4′′a), 105.49 (C-4′′a), 118.87 (C-4′′′a), 118.89 (C-4′′′a), 123.74 (C-3′), 123.76 (C-3′), 124.38 (C-5′′′), 124.40 (C-5′′′), 126.03 (C-6′′′), 126.10 (C-6′′′), 126.41 (C-3′′), 126.47 (C-3′′), 127.69 (C-8′′′), 127.72 (C-8′′′), 128.41 (C-8′′), 128.63 (C-5′′), 128.64 (C-5′′), 129.17 (C-6′′), 129.18 (C-6′′), 129.57 (C-5′), 129.66 (C-5′), 130.00 (C-4′a), 130.06 (C-4′a), 133.51 (C-4′), 133.53 (C-4′), 135.11 (C-8′a), 135.16 (C-8′a), 136.39 (C-7′′′), 136.45 (C-7′′′), 136.75 (C-7′′), 136.76 (C-7′′), 145.72 (C-2′), 145.75 (C-2′), 146.89 (C-4′′), 146.92 (C-4′′), 147.96 (C-8′), 148.04 (C-8′), 149.72 (C-8′′a), 149.73 (C-8′′a), 149.78 (C-8′′′a), 152.27 (C-2′′), 152.29 (C-2′′), 152.37 (C-2′′′), 152.43 (C-2′′′), 152.75 (C-4′′′), 152.81 (C-4′′′), 157.71 (C-6′), 157.75 (C-6′) ppm. MS (EI, 70 eV): m/z (%) = 583.1/582.1/581.1 [M]^+•^ (14/13/7), 406.1/405.1/404.1 [M-C_9_H_6_ClN_2_]^+^ (18/45/82), 403.1/402.1 [M-C_10_H_10_ClN]^+•^ (95/100), 365.1/364.1/363.1/362.1/361.1/360.1 [C_21_H_16_ClN_3_O]^+•^ (17/30/54/67/23/17), 349.1/348.1/347.1 [C_20_H_14_ClN_3_O]^+•^ (15/23/33), 337.1/336.1/335.1 [C_19_H_14_ClN_3_O]^+•^ (13/18/17), 248.1/247.1/246.1 [C_14_H_15_ClN_2_]^+•^ (14/20/23), 208.1/207.1/206.1/205.1 [C_11_H_10_ClN_2_]^+^ (14/25/37/37), 194.1/193.1/192.1/191.1 [C_10_H_8_ClN_2_]^+^ (13/22/26/20), 182.1/181.1/180.1/179.1/178.1 [C_9_H_7_ClN_2_]^+•^ (20/36/56/52/20), HRMS (ESI) calcd. [M+H]^+^ 582.18219; found 582.18207.

#### 3.2.2. 6-Methoxyquinolin-8-amine (**4**)

A suspension of the commercially available 6-methoxy-8-nitroquinoline (**7**, 1.830 g, 8.96 mmol) and Pd/C (183.0 mg, 10% m/m) in MeOH (dry, 50 mL) was stirred under hydrogen atmosphere at room temperature for 2 hours. The reaction mixture was filtered (Celite) and concentrated, and the residue was purified by flash column chromatography on deactivated silica gel (petroleum ether/ethyl acetate 5 : 1). Compound** 4** was obtained as yellow oil (1.530 g, 98%). IR (ATR-FTIR): $\tilde{v}=3363$ (w, br), 2933 (w, br), 2360 (w), 2341 (w), 1616 (s), 1589 (s), 1577 (m), 1502 (s), 1467 (m), 1451 (m), 1425 (m), 1381 (s), 1336 (m), 1275 (w), 1214 (m), 1196 (m), 1158 (s), 1082 (m), 1051 (m), 1028 (m), 977 (w), 956 (w), 898 (w), 886 (w), 820 (s), 789 (s), 752 (w), 738 (w), 652 (m), 635 (m), 622 (w), 611 (w) cm^−1^. ^1^H-NMR (600 MHz, MeOD-d_4_): *δ* = 3.85 (s, 3 H, OMe), 6.56 (d, ^4^ *J*_H–H_ = 2.64 Hz, 1 H), 6.61 (d, ^4^ *J*_H–H_ = 2.58 Hz, 1 H), 7.35 (dd, ^3^ *J*_H–H_ = 4.20 Hz, 8.28 Hz, 1 H, 3-H), 8.04 (dd, ^3^ *J*_H–H_ = 8.28 Hz, ^4^ *J*_H–H_ = 1.62 Hz, 1 H, 4-H), 8.52 (dd, ^3^ *J*_H–H_ = 4.20 Hz, ^4^ *J*_H–H_ = 1.62 Hz, 1 H, 2-H) ppm. ^13^C-NMR (150 MHz, MeOD-d_4_): *δ* = 55.81 (6-OMe), 95.59 (CH), 103.15 (CH), 122.91 (CH), 131.75 (Cq), 136.57 (CH), 136.64 (Cq), 146.01 (CH), 147.03 (C-8), 160.67 (C-6) ppm. MS (EI, 70 eV): m/z (%) = 175.1/174.1 [M]^+•^ (3/25), HRMS (ESI) calcd. [M+H]^+^ 175.08659; found 175.08658.

#### 3.2.3. 7-Chloro-*N*-(6′-methoxyquinolin-8′-yl)quinolin-4-amine (**9**)

A total of 49.5 mg (0.28 mmol) of 6-methoxy-8-aminoquinoline (**4**) and 38.3 mg (0.19 mmol) of 4,7-dichloroquinoline were allowed to react at 120°C under neat conditions for 9 hours. The mixture was allowed to cool down; the residue was suspended in dichloromethane and methanol and finally alkalized with a small amount of ammonia solution. The solvent mixture was removed under reduced pressure; the residue was suspended in dichloromethane, filtered (Celite), and concentrated. Purification on deactivated silica gel (petroleum ether/ethyl acetate 5 : 1) gave product** 9** (60.5 mg, 95%) as beige crystals. Mp 209°C (petroleum ether/ethyl acetate), IR (ATR-FTIR): $\tilde{v}=3726$ (w), 3628 (w), 3347 (w), 3029 (w), 2981 (w), 2947 (w), 2360 (m), 2341 (m), 2164 (w), 1888 (w), 1626 (w), 1565 (s), 1536 (s), 1499 (m), 1459 (m), 1445 (m), 1428 (m), 1395 (m), 1364 (m), 1348 (m), 1326 (m), 1273 (w), 1254 (w), 1213 (m), 1196 (m), 1159 (m), 1146 (m), 1120 (w), 1096 (w), 1072 (m), 1051 (w), 1038 (w), 1028 (w), 993 (w), 959 (w), 909 (w), 893 (w), 867 (m), 848 (m), 825 (m), 802 (m), 785 (m), 772 (w), 758 (w), 690 (w), 669 (w), 650 (w), 633 (w), 617 (w), 609 (w) cm^−1^. ^1^H-NMR (600 MHz, CD_2_Cl_2_): *δ* = 3.95 (s, 3 H, OMe), 6.78 (d, ^4^ *J*_H–H_ = 2.40 Hz, 1 H, 5′-H), 7.43 (d, ^4^ *J*_H–H_ = 2.40 Hz, 1 H, 7′-H), 7.48 (dd, ^3^ *J*_H–H_ = 4.20 Hz, 8.22 Hz, 1 H, 3′-H), 7.55 (dd, ^3^ *J*_H–H_ = 9.00 Hz, ^4^ *J*_H–H_ = 2.10 Hz, 1 H, 6-H), 7.59 (d, ^3^ *J*_H–H_ = 5.10 Hz, 1 H, 3-H), 8.05 (d, ^4^ *J*_H–H_ = 1.98 Hz, 1 H, 8-H), 8.12 (dd, ^3^ *J*_H–H_ = 8.28 Hz, ^4^ *J*_H–H_ = 1.38 Hz, 1 H, 4′-H), 8.20 (d, ^3^ *J*_H–H_ = 8.88 Hz, 1 H, 5-H), 8.71–8.73 (m, 2 H, 2-H, 2′-H), 9.48 (s, 1 H, NH) ppm. ^13^C-NMR (150 MHz, CD_2_Cl_2_): *δ* = 56.82 (OMe), 97.99 (C-5′), 105.28 (C-7′), 105.37 (C-3), 120.45 (C-4a), 122.83 (C-5), 123.15 (C-3′), 126.89 (C-6), 129.48 (C-8), 130.34 (C-4′a), 135.65 (C-7), 135.73 (C-4′), 136.55 (C-8′), 138.44 (C-8′a), 145.38 (C-4), 146.28 (C-2′), 150.53 (C-8a), 152.71 (C-2), 158.89 (C-6′) ppm. MS (EI, 70 eV): m/z (%) = 338.2/337.2/336.2/335.2 [M]^+•^ (7/33/37/100), HRMS (ESI) calcd. [M+H]^+^ 336.08982; found 336.08964.

#### 3.2.4. 7-Chloro-*N*-(7′′-chloroquinolin-4′′-yl)-*N*-(6′-methoxyquinolin-8′-yl)quinolin-4-amine (**10**)

6-Methoxy-8-aminoquinoline (**4**, 62.1 mg, 0.36 mmol) and 4,7-dichloroquinoline (212.8 mg, 1.07 mmol) were heated at 120°C under neat conditions for 9 hours. The reaction mixture was suspended in dichloromethane; methanol was added, and the mixture was alkalized by the addition of ammonia solution. The solvent mixture was removed under reduced pressure; the residue was suspended in dichloromethane, filtered (Celite), and concentrated. Purification by flash column chromatography on deactivated silica gel (gradient elution starting with petroleum ether/ethyl acetate 6 : 1, followed by petroleum ether/ethyl acetate 5 : 1) yielded compound** 10** (45.4 mg, 25%) as yellow solid. Mp 135–140°C (petroleum ether/ethyl acetate), IR (ATR-FTIR): $\tilde{v}=3159$ (w, br), 2925 (w), 2853 (w), 2461 (w), 2194 (w, br), 2059 (w), 1609 (m), 1563 (s), 1498 (s), 1473 (m), 1455 (m), 1433 (m), 1386 (m), 1356 (m), 1326 (m), 1294 (w), 1267 (w), 1237 (w), 1218 (m), 1186 (w), 1159 (w), 1139 (w), 1108 (m), 1073 (m), 1037 (w), 982 (w), 959 (w), 916 (m), 877 (m), 838 (m), 826 (m), 815 (m), 794 (m), 781 (m), 755 (w), 732 (w), 698 (w), 682 (w), 657 (w), 635 (w), 613 (w) cm^−1^. ^1^H-NMR (600 MHz, MeOD-d_4_/CD_2_Cl_2_): *δ* = 3.84 (s, 3 H, OMe), 7.39 (dd, ^3^ *J*_H–H_ = 4.02 Hz, 8.64 Hz, 1 H, 3′-H), 7.42–7.46 (m, 2 H, 5′′-H, 6′′-H), 7.50 (d, ^3^ *J*_H–H_ = 4.44 Hz, 1 H, 3′′-H), 7.56 (dd, ^3^ *J*_H–H_ = 8.64 Hz, ^4^ *J*_H–H_ = 1.44 Hz, 1 H, 4′-H), 7.66 (dd, ^3^ *J*_H–H_ = 8.88 Hz, ^4^ *J*_H–H_ = 2.04 Hz, 1 H, 6-H), 7.72 (d, ^3^ *J*_H–H_ = 5.40 Hz, 1 H, 3-H), 7.84 (s, 1 H, 7′-H), 8.04 (d, ^4^ *J*_H–H_ = 2.04 Hz, 1 H, 8-H), 8.16 (d, ^4^ *J*_H–H_ = 1.68 Hz, 1 H, 8′′-H), 8.38 (d, ^3^ *J*_H–H_ = 8.88 Hz, 1 H, 5-H), 8.71 (d, ^3^ *J*_H–H_ = 5.34 Hz, 1 H, 2-H), 8.78 (dd, ^3^ *J*_H–H_ = 4.02 Hz, ^4^ *J*_H–H_ = 1.50 Hz, 1 H, 2′-H), 9.00 (d, ^3^ *J*_H–H_ = 4.38 Hz, 1 H, 2′′-H) ppm. 5′-H was not detectable because of the proton-deuterium exchange at this position. ^13^C-NMR (150 MHz, MeOD-d_4_/CD_2_Cl_2_): *δ* = 57.23 (OMe), 102.63 (C-7′), 105.92 (C-3), 113.14 (C-5′), 121.07 (C-4a), 124.02 (C-5), 124.23 (C-3′), 125.67 (C-3′′), 127.93 (C-6), 128.30 (C-4′′a), 128.54 (C-8), 128.58 (C-8′′), 128.90 (C-5′′, C-6′′), 129.91 (C-4′a), 134.16 (C-4′), 136.43 (C-8′a), 136.89 (C-7′′), 137.20 (C-7), 140.29 (C-8′), 145.17 (C-8′′a), 147.56 (C-8), 147.69 (C-2′), 149.48 (C-4′′), 150.34 (C-4), 152.06 (C-2′′), 152.87 (C-2), 156.13 (C-6′) ppm. MS (EI, 70 eV): m/z (%) = 500.9/499.9/498.9/497.9/496.9/495.9 [M]^+•^ (6/17/34/73/62/100), HRMS (ESI) calcd. [M+H]^+^ 497.09304; found 497.09284.

#### 3.2.5. 3-Bromopropanoyl Chloride (**19**) [60]

3-Bromopropionic acid (5.36 g, 35.04 mmol) was refluxed in thionyl chloride (10 mL) under nitrogen atmosphere. The complete conversion was determined by ^1^H-NMR spectroscopy. The excess of thionyl chloride was removed by distillation, and compound** 19** (3.76 g, 63%) was obtained as yellow oil. IR (ATR-FTIR): $\tilde{v}=1787$ (s), 1742 (m), 1430 (w), 1391 (w), 1345 (w), 1327 (w), 1266 (m), 1215 (w), 1159 (w), 1034 (m), 1016 (m), 954 (s), 907 (m), 871 (m), 842 (m), 764 (m), 751 (m), 679 (s), 642 (w), 631 (w), 603 (s) cm^−1^. ^1^H-NMR (600 MHz, CDCl_3_): *δ* = 3.44 (t, ^3^ *J*_H–H_ = 6.26 Hz, 2 H, CH_2_), 3.53 (t, ^3^ *J*_H–H_ = 6.44 Hz, 2 H, CH_2_) ppm. ^13^C-NMR (150 MHz, CDCl_3_): *δ* = 24.00 (CH_2_), 49.38 (CH_2_), 171.45 (COCl) ppm. MS (EI, 70 eV): m/z (%) = 137.0/136.0/135.0 [M-Cl]^+^ (93/4/100), 109.0/108.0/107.0 [M-CClO]^+^ (48/5/49).

#### 3.2.6. *tert*-Butyl Piperazine-1-carboxylate (**20**) [74]

A solution of Boc_2_O (5.471 g, 25.07 mmol, 0.5 mM) in DCM (dry, 50 mL) was slowly dropped to a solution of piperazine (4.323 g, 50.14 mmol, 0.4 mM) in DCM (dry, 125 mL) at 0°C for 70 min and was stirred for 1 hour at 0°C. The solvent was removed under reduced pressure, and the obtained colorless residue was suspended in 75 mL of water. The double protected and colorless compound** 22** was filtered off; the filtrate was alkalized using aqueous K_2_CO_3_ solution and exhaustively extracted with diethyl ether. The combined organic extracts were dried (MgSO_4_), filtered, and concentrated. Purification with flash column chromatography on deactivated silica gel (DCM/MeOH 10 : 1) and recrystallisation (diethyl ether) gave** 20** (3.653 g, 78%) as colorless crystals. Mp 44°C (diethyl ether), IR (ATR-FTIR): $\tilde{v}$ = 3003–2733 (w, br), 1685 (s), 1475 (w), 1455 (w), 1421 (s), 1361 (m), 1339 (w), 1316 (m), 1291 (m), 1268 (m), 1243 (s), 1167 (s), 1139 (m), 1119 (s), 1090 (m), 1052 (m), 1005 (s), 926 (w), 902 (w), 865 (m), 846 (w), 809 (m), 765 (s), 655 (w), 621 (w), 602 (w) cm^−1^. ^1^H-NMR (600 MHz, CDCl_3_): *δ* = 1.43 (s, 9 H,* t*Bu-Me), 1.95 (s, 1 H, NH), 2.78 (m, 4 H, 3-CH_2_, 5-CH_2_), 3.36 (m, 4 H, 2-CH_2_, 6-CH_2_) ppm. ^13^C-NMR (150 MHz, CDCl_3_): *δ* = 28.63 (*t*Bu-Me), 44.32 (br, CH_2_), 45.40 (br, CH_2_), 46.05 (C-3, C-5), 79.80 (*t*Bu-C), 155.03 (Boc CO) ppm. MS (EI, 70 eV): m/z (%) = 187.1/186.1 [M]^+•^ (2/17), 131.0/130.0 [M-C_4_H_8_]^+•^ (6/46), 114.0/113.0 [M-C_4_H_9_O]^+^ (2/37), 86.0/85.0 [C_4_H_9_N_2_]^+^ (3/24), 58.0/57.0 [C_4_H_9_]^+^ (7/100), HRMS (ESI) calcd. [M+H]^+^; found 187.14410.

#### 3.2.7. Di-*tert*-Butyl Piperazine-1,4-dicarboxylate (**22**)

Compound** 18** was obtained as side product within the synthesis of** 20** and separated by filtration. For analysis, it was further purified by flash column chromatography on deactivated silica gel (DCM/MeOH 100 : 1) and gave compound** 22** (612.2 mg, 9%) as colorless solid. Mp 111°C (DCM/MeOH), IR (ATR-FTIR): $\tilde{v}=2983$ (w, br), 2863 (w, br), 2363 (w), 2336 (w), 1682 (s), 1482 (w), 1455 (m), 1417 (s), 1362 (s), 1289 (m), 1239 (s), 1215 (w), 1159 (s), 1105 (s), 1005 (s), 972 (m), 865 (s), 835 (w), 768 (s), 669 (w) cm^−1^. ^1^H-NMR (600 MHz, CDCl_3_): *δ* = 1.44 (s, 18 H,* t*Bu-Me), 3.36 (s, 8 H, piperazine CH_2_) ppm. ^13^C-NMR (150 MHz, CDCl_3_): *δ* = 28.60 (*t*Bu-Me), 43.67 (b, piperazine CH_2_), 80.24 (*t*Bu-C), 154.91 (Boc CO) ppm. MS (EI, 70 eV): m/z (%) = 286.1 [M]^+•^ (3), 175.0/174.0 [C_6_H_10_N_2_O_4_]^+•^ (3/28), 58.0/57.0 [C_4_H_9_]^+^ (5/100), HRMS (ESI) calcd. [M+Na]^+^ 309.17848; found 309.17847 [M+Na]^+^.

#### 3.2.8. *tert*-Butyl 4-(3′-Bromopropanoyl)piperazine-1-carboxylate (**21**)

A solution of 3-bromopropionyl chloride (**19**, 176.0 mg, 1.03 mmol) was slowly dropped to a suspension of the monoprotected piperazine** 20** (95.7 mg, 0.51 mmol) and of sodium acetate (105.7 mg, 1.29 mmol) in DCM (dry, 3 mL) at 0°C. After completed addition, the reaction mixture was allowed to warm up to room temperature, and the full conversion was determined by thin layer chromatography (deactivated silica gel DCM/MeOH 10 : 1). The excess of sodium acetate was filtered off (Celite) and all volatile substances were removed under reduced pressure. Purification by flash column chromatography was obtained on silica gel (DCM/diethyl ether 5 : 1) and gave compound** 21** (149.6 mg, 91%) as colorless crystals. Mp 102°C (DCM/diethyl ether), IR (ATR-FTIR): $\tilde{v}=2976$ (w), 2920 (w, br), 1679 (s), 1635 (s), 1455 (m), 1426 (s), 1402 (s), 1361 (s), 1277 (m), 1263 (m), 1239 (s), 1176 (s), 1129 (s), 1071 (s), 1057 (m), 1012 (s), 993 (m), 956 (m), 930 (m), 909 (m), 889 (w), 868 (m), 843 (w), 824 (w), 762 (s), 721 (w), 647 (w), 615 (w) cm^−1^. ^1^H-NMR (600 MHz, CDCl_3_): *δ* = 1.44 (s, 9 H,* t*Bu-Me), 2.90 (t, ^3^ *J*_H–H_ = 7.08 Hz, 2 H, 2′-CH_2_), 3.38–3.46 (m, br, 6 H, piperazine CH_2_), 3.57–3.60 (m, br, 2 H, piperazine CH_2_), 3.63 (t, ^3^ *J*_H–H_ = 7.08 Hz, 2 H, 3′-CH_2_) ppm. ^13^C-NMR (150 MHz, CDCl_3_): *δ* = 27.28 (C-3′), 28.57 (*t*Bu-Me), 36.49 (C-2′), 41.79 (CH_2_), 43.73 (br, CH_2_), 45.50 (CH_2_), 80.67 (*t*Bu-C), 154.73 (Boc CO), 168.91 (amide CO) ppm. MS (EI, 70 eV): m/z (%) = 322.0/320.0 [M]^+•^ (1/1), 266.0/265.0/264.0 [M-C_4_H_8_]^+•^ (7/4/8), 86.0/85.0 [C_4_H_9_N_2_]^+^ (2/17), 58.0/57.0 [C_4_H_9_]^+^ (5/100), HRMS (ESI) [M+Na]^+^ 343.06278; found 343.06278.

#### 3.2.9. *tert*-Butyl 4-Acryloylpiperazine-1-carboxylate (Not Displayed)

The compound was obtained as side product within the synthesis of compound** 21** and purified for analysis by flash column chromatography on silica gel (DCM/diethyl ether 5 : 1) to give colorless crystals. Mp 85°C (DCM/diethyl ether) IR (ATR-FTIR): $\tilde{v}=2970$ (w), 2925 (w), 2862 (w), 2361 (m), 2340 (m), 1691 (s), 1640 (s), 1608 (m), 1520 (w), 1455 (m), 1416 (s), 1362 (m), 1282 (m), 1250 (s), 1227 (m), 1162 (s), 1127 (s), 1083 (m), 1054 (m), 1030 (s), 997 (m), 980 (m), 948 (m), 927 (m), 862 (m), 841 (w), 828 (w), 788 (m), 765 (m), 668 (w) cm^−1^. ^1^H-NMR (600 MHz, CDCl_3_): *δ* = 1.45 (s, 9 H,* t*Bu-Me), 3.43–3.64 (m, 8 H, 2-CH_2_, 3-CH_2_, 5-CH_2_, 6-CH_2_), 5.70 (dd, ^2^ *J*_H–H_ = 1.62 Hz, ^3^ *J*_H–H_ = 10.62 Hz, 1 H, 3′-H), 6.29 (dd, ^2^ *J*_H–H_ = 1.62 Hz, ^3^ *J*_H–H_ = 16.74 Hz, 1 H, 3′-H), 6.53 (dd, ^3^ *J*_H–H_ = 10.62 Hz, 16.74 Hz, 1 H, 2′-H) ppm. ^13^C-NMR (150 MHz, CDCl_3_): *δ* = 29.58 (*t*Bu-Me), 41.98 (CH_2_), 43.85 (br, CH_2_), 45.82 (CH_2_), 80.58 (*t*Bu-C), 127.45 (C-2′), 128.57 (C-3′), 154.76 (Boc CO), 165.77 (amide CO) ppm. MS (EI, 70 eV): m/z (%) = 241.1/240.1 [M]^+•^ (2/13), 185.0/184.0 [M-C_4_H_8_]^+^ (3/28), 113.0 [C_5_H_9_N_2_O]^+^ (13), 58.0/57.0 [C_4_H_9_]^+^ (6/100), HRMS (ESI) calcd. [M+Na]^+^ 263.13661; found 263.13661.

#### 3.2.10. *tert*-Butyl 4-(3′-(6′′-Methoxyquinolin-8′′-ylamino)propanoyl)piperazine-1-carboxylate (**23**)

NaH (782.6 mg of a 55% oily dispersion, 17.93 mmol) was added in portions to a solution of 6-methoxy-8-aminoquinoline (**4**, 1.041 g, 5.97 mmol) in DMF (dry, 30 mL) under nitrogen atmosphere at 0°C. The reaction mixture was stirred for 30 min at 0°C, followed by the addition of the bromine derivative** 21** in portions. The mixture was stirred for 1 hour at 0°C, allowed to warm up to room temperature, and stirred for further 18 hours. The excess of NaH of the reddish brown suspension was carefully hydrolyzed by a small amount of water. The solvent DMF was azeotropically removed using toluene under reduced pressure; the residue was suspended in dichloromethane and filtered (Celite). The filtrate was concentrated and purified by flash column chromatography on silica gel (ethyl acetate 100%). The obtained light yellowish foam was recrystallized (diethyl ether/petroleum ether) and gave compound** 23** (1.838 g, 74%) as light yellow crystals. Mp 45°C (petroleum ether/ethyl acetate), IR (ATR-FTIR): $\tilde{v}$ = 2975–2858 (w, br), 2359 (m), 2341 (w), 1689 (m), 1644 (m), 1616 (m), 1594 (w), 1577 (w), 1518 (m), 1455 (m), 1417 (m), 1388 (m), 1363 (m), 1283 (w), 1255 (m), 1236 (m), 1213 (m), 1197 (m), 1161 (s), 1123 (m), 1078 (w), 1050 (w), 1024 (m), 995 (m), 904 (w), 862 (w), 820 (m), 790 (m), 765 (m), 667 (w), 643 (w), 627 (w), 615 (m), 602 (w) cm^−1^. ^1^H-NMR (600 MHz, MeOD-d_4_): *δ* = 1.44 (s, 9 H,* t*Bu-Me), 2.80 (t, ^3^ *J*_H–H_ = 6.36 Hz, 2 H, 2′-CH_2_), 3.28-3.29 (m, 2 H, CH_2_), 3.32–3.36 (m, 2 H, CH_2_), 3.46–3.48 (m, 2 H, CH_2_), 3.55-3.56 (m, 2 H, CH_2_), 3.63 (t, ^3^ *J*_H–H_ = 6.36 Hz, 2 H, 3′-CH_2_), 3.87 (s, 3 H, OMe), 6.38 (d, ^4^ *J*_H–H_ = 2.46 Hz, 1 H, 7′′-H), 6.48 (d, ^4^ *J*_H–H_ = 2.52 Hz, 1 H, 5′′-H), 7.35 (dd, ^3^ *J*_H–H_ = 4.26 Hz, 8.28 Hz, 3′′-H), 8.02 (dd, ^3^ *J*_H–H_ = 8.28 Hz, ^4^ *J*_H–H_ = 1.50 Hz, 1 H, 4′′-H), 8.49 (dd, ^3^ *J*_H–H_ = 4.14, ^4^ *J*_H–H_ = 1.62 Hz, 1 H, 2′′-H) ppm. ^13^C-NMR (150 MHz, MeOD-d_4_): *δ* = 28.73 (*t*Bu-Me), 33.32 (C-2′), 40.41 (C-3′), 42.76 (CH_2_), 43.90 (b, CH_2_), 45.09 (b, CH_2_), 46.69 (CH_2_), 55.83 (OMe), 81.73 (*t*Bu-C), 93.77 (C-5′′), 98.34 (C-7′′), 123.17 (C-3′′), 131.63 (C-4′′a), 136.32 (C-4′′), 136.73 (C-8′′a), 145.74 (C-2′′), 146.78 (C-8′′), 156.37 (Boc CO), 161.08 (C-6′′), 172.95 (C-1′) ppm. MS (EI, 70 eV): m/z (%) = 416.2/415.2/414.2 [M]^+•^ (3/16/42), 203.1/202.1/201.1 [C_12_H_13_N_2_O]^+^ (2/16/59), 188.1/187.1 [C_11_H_11_N_2_O]^+^ (14/100), 175.1/174.1 [C_10_H_10_N_2_O]^+•^ (10/24), HRMS (ESI) calcd. [M]^+•^ 414.22616; found 414.22615.

#### 3.2.11. 1-(3-(4-(*tert*-Butoxycarbonyl)piperazin-1-yl)-3-oxopropyl)-8-(3-(4-(*tert*-butoxycarbonyl)piperazin-1-yl)-3-oxopropylamino)-6-methoxyquinolinium Bromide (**24**)

Further elution of the column chromatography of compound** 23** (ethyl acetate 100%) and recrystallization (DCM/petroleum ether) gave the side product** 24** (684.1 mg, 1.04 mmol, 17%) as beige crystals. Mp 168°C (DCM/petroleum ether), IR (ATR-FTIR): $\tilde{v}=3734$ (w), 3627 (w), 3385 (w), 2978–2861 (w, br), 2360 (m), 2341 (w), 1704 (m), 1688 (m), 1647 (m), 1626 (s), 1580 (w), 1524 (m), 1455 (m), 1420 (s), 1389 (m), 1364 (m), 1283 (w), 1251 (m), 1221 (m), 1163 (s), 1122 (m), 1075 (w), 1019 (m), 996 (m), 932 (w), 902 (w), 862 (w), 823 (m), 790 (m), 766 (w), 669 (w), 650 (w), 632 (w), 620 (w), 610 (w) cm^−1^. ^1^H-NMR (600 MHz, CD_2_Cl_2_): *δ* = 1.39 (s, 9 H,* t*Bu-Me), 1.44 (s, 9 H,* t*Bu-Me), 1.84–1.90 (m, 1 H, CH_2_), 2.00–2.06 (m, 1 H, CH_2_), 2.24–2.29 (m, 1 H, CH_2_), 2.40–2.45 (m, 1 H, CH_2_), 2.96–3.05 (m, 1 H, piperazine CH_2_), 3.16–3.27 (m, 2 H, piperazine CH_2_), 3.33–3.64 (m, 17 H, 1′-CH_2_, 1′′′-CH_2_, piperazine CH_2_), 3.87 (s, 3 H, OMe), 6.30 (d, ^3^ *J*_H–H_ = 2.46 Hz, 1 H, 7-H), 6.38 (d, ^3^ *J*_H–H_ = 2.46 Hz, 1 H, 5-H), 6.43 (t, ^3^ *J*_H–H_ = 6.06 Hz, 1 H, NH), 7.31 (dd, ^3^ *J*_H–H_ = 4.14 Hz, 8.28 Hz, 1 H, 3-H), 7.94 (dd, ^3^ *J*_H–H_ = 8.28 Hz, ^4^ *J*_H–H_ = 1.62 Hz, 1 H, 4-H), 8.51 (dd, ^3^ *J*_H–H_ = 4.14 Hz, ^4^ *J*_H–H_ = 1.62 Hz, 1 H, 2-H) ppm. ^13^C-NMR (150 MHz, CD_2_Cl_2_): *δ* = 26.37 (CH_2_), 28.54 (*t*Bu-Me), 28.59 (*t*Bu-Me), 30.74 (CH_2_), 41.80 (NCH_2_), 42.23 (NCH_2_), 43.57 (br, piperazine CH_2_), 44.43 (br, piperazine CH_2_), 45.74 (piperazine CH_2_), 45.98 (piperazine CH_2_), 46.06 (piperazine CH_2_), 55.71 (OMe), 80.21 (*t*Bu-C), 80.31 (*t*Bu-C), 92.71 (C-5), 96.94 (C-7), 122.53 (C-3), 130.35 (C-4a), 135.20 (C-4), 135.68 (C-8a), 145.08 (C-2), 145.91 (C-8), 154.82 (Boc CO), 154.87 (Boc CO), 159.90 (C-6), 171.10 (amide CO), 173.38 (amide CO) ppm. ^15^N-NMR: (40.5 MHz, DMSO-d_6_,): 62 (ArNH), 82 (amide-N), 115 (Boc-N), 119 (Boc-N), 292 (ArN^+^) ppm. MS (EI, 70 eV): m/z (%) = 654.3 [M]^+•^ (3), 555.3/554.3 [M-C_5_H_8_O_2_]^+•^ (6/17), 455.2/454.2 [M-2(C_5_H_8_O_2_)]^+•^ (5/15), 188.1/187.1 [C_11_H_11_N_2_O]^+^ (15/100), 175.1/174.1 [C_10_H_10_N_2_O]^+•^ (7/19), HRMS (ESI) calcd. [M+Na]^+^ 677.36332; found 677.36340.

#### 3.2.12. 3-(6′′-Methoxyquinolin-8′′-ylamino)-1-(piperazin-1′-yl)propan-1-one (**12**)

TFA (3.2 mL, 0.04 mmol) was added to a yellowish solution of the Boc protected derivative** 23** (352.4 mg, 0.85 mmol) in DCM (12 mL) at 25°C, leading to a change of color to an intense orange color. The reaction mixture was stirred for 1 hour at 25°C, followed by the addition of aqueous NaHCO_3_ solution (decolorization of the solution) and the exhaustive extraction using dichloromethane. The combined organic extracts were dried (MgSO_4_) and filtered, and all volatile components were removed under reduced pressure. Purification with flash column chromatography on deactivated silica gel (DCM/MeOH 10 : 1) gave compound** 12** (262.0 mg, 98%) as yellow solid. Mp 75°C (DCM/MeOH), IR (ATR-FTIR): $\tilde{v}=3376$ (w, br), 2916 (w, br), 2856 (w, br), 2361 (m), 2342 (w), 1614 (s), 1576 (m), 1517 (s), 1446 (m), 1422 (m), 1386 (m), 1336 (w), 1320 (w), 1265 (w), 1237 (w), 1212 (m), 1196 (m), 1165 (m), 1153 (m), 1123 (w), 1048 (w), 1024 (m), 906 (w), 820 (s), 790 (s), 668 (m), 652 (m), 634 (m), 615 (w) cm^−1^. ^1^H-NMR (600 MHz, MeOD-d_4_): *δ* = 2.73-2.74 (m, 2 H, piperazine CH_2_), 2.78-2.79 (m, 4 H, 2-CH_2_, piperazine CH_2_), 3.49–3.51 (m, 2 H, piperazine CH_2_), 3.58–3.60 (m, 2 H, piperazine CH_2_), 3.62 (t, ^3^ *J*_H–H_ = 6.42 Hz, 2 H, 3-CH_2_), 3.87 (s, 3 H, OMe), 6.38 (d, ^4^ *J*_H–H_ = 2.40 Hz, 1 H, 7′′-H), 6.48 (d, ^4^ *J*_H–H_ = 2.40 Hz, 1 H, 5′′-H), 7.35 (dd, ^3^ *J*_H–H_ = 4.26 Hz, 8.28 Hz, 1 H, 3′′-H), 8.01 (dd, ^3^ *J*_H–H_ = 8.28 Hz, ^4^ *J*_H–H_ = 1.50 Hz, 1 H, 4′′-H), 8.50 (dd, ^3^ *J*_H–H_ = 4.26 Hz, ^4^ *J*_H–H_ = 1.50 Hz, 1 H, 2′′-H) ppm. ^13^C-NMR (150 MHz, MeOD-d_4_): *δ* = 33.20 (C-2), 40.41 (C-3), 40.45 (C-3), 42.99 (CH_2_), 46.01 (CH_2_), 46.35 (CH_2_), 47.15 (CH_2_), 55.82 (OMe), 93.77 (C-5′′), 98.31 (C-7′′), 123.16 (C-3′′), 131.62 (C-4′′a), 136.31 (C-4′′), 136.73 (C-8′′a), 145.75 (C-2′′), 146.80 (C-8′′), 146.81 (C-8′′), 172.71 (amide CO), 172.73 (amide CO) ppm. MS (EI, 70 eV): m/z (%) = 316.2/315.2/314.2 [M]^+•^ (5/20/41), 202.1/201.1 [C_12_H_13_N_2_O]^+^ (19/48), 188.1/187.1 [C_11_H_11_N_2_O]^+^ (19/100), 175.1/174.1 [C_10_H_10_N_2_O]^+•^ (14/29), HRMS (ESI) calcd. [M+H]^+^ 315.18155; found 315.18155.

#### 3.2.13. 1-(4′′-(7′′′-Chloroquinolin-4′′′-yl)piperazin-1′′-yl)-3-(6′-methoxyquinolin-8′-ylamino)propan-1-one (**13**)

Compound** 12** (71.7 mg, 0.23 mmol) and 4,7-dichloroquinoline (45.5 mg, 0.23 mmol) were allowed to react at 120°C under neat conditions for 4.5 hours. The residue was suspended in methanol, the reddish brown mixture was alkalized using ammonia solution (change of color to yellow), and all volatile components were removed under reduced pressure. Purification by flash column chromatography on deactivated silica gel (ethyl acetate 100%) gave compound** 11** (67.5 mg, 62%) as light yellow solid. Mp 85°C (ethyl acetate), IR (ATR-FTIR): $\tilde{v}=3383$ (w), 2920 (w), 2853 (w), 2360 (m), 2341 (m), 1638 (s), 1615 (s), 1574 (s), 1517 (s), 1456 (m), 1421 (s), 1382 (s), 1334 (w), 1276 (w), 1212 (m), 1196 (m), 1154 (m), 1124 (m), 1070 (w), 1049 (w), 1011 (m), 933 (w), 909 (w), 866 (m), 819 (s), 790 (m), 714 (w), 669 (w), 654 (w), 628 (w) cm^−1^. ^1^H-NMR (600 MHz, MeOD-d_4_): *δ* = 2.87 (t, ^3^ *J*_H–H_ = 6.36 Hz, 2 H, 2-CH_2_), 3.01–3.03 (m, 2 H, piperazine CH_2_), 3.11-3.12 (m, 2 H, piperazine CH_2_), 3.70 (t, ^3^ *J*_H–H_ = 6.30 Hz, 1 H, 3-CH_2_), 3.78-3.79 (m, 2 H, piperazine CH_2_), 3.87-3.88 (m, 5 H, piperazine CH_2_, OMe), 6.42 (d, ^4^ *J*_H–H_ = 2.40 Hz, 1 H, 7′-H), 6.48 (d, ^4^ *J*_H–H_ = 2.40 Hz, 1 H, 5′-H), 7.35 (dd, ^3^ *J*_H–H_ = 4.14 Hz, 8.28 Hz, 1 H, 3′-H), 7.52 (dd, ^3^ *J*_H–H_ = 8.94 Hz, ^4^ *J*_H–H_ = 2.10 Hz, 1 H, 6′′′-H), 7.93 (d, ^4^ *J*_H–H_ = 2.10 Hz, 1 H, 8′′′-H), 8.01 (dd, ^3^ *J*_H–H_ = 8.28 Hz, ^4^ *J*_H–H_ = 1.62 Hz, 1 H, 4′-H), 8.04 (d, ^3^ *J*_H–H_ = 8.94 Hz, 1 H, 5′′′-H), 8.49 (dd, ^3^ *J*_H–H_ = 4.14 Hz, ^4^ *J*_H–H_ = 1.62 Hz, 1 H, 2′-H), 8.61 (d, ^3^ *J*_H–H_ = 5.10 Hz, 1 H, 2′′′-H) ppm. ^13^C-NMR (150 MHz, MeOD-d_4_): *δ* = 33.25 (C-2), 40.50 (C-3), 42.98 (piperazine CH_2_), 47.01 (piperazine CH_2_), 53.16 (piperazine CH_2_), 53.21 (piperazine CH_2_), 55.85 (OMe), 93.78 (C-5′), 98.36 (C-7′), 110.74 (C-3′′′), 123.21 (C-3′), 123.23 (C-4′′′a), 127.12 (C-5′′′), 127.75 (C-6′′′), 128.51 (C-8′′′), 131.68 (C-4′a), 136.35 (C-4′), 136.74 (C-8′a), 136.77 (C-7′′′), 145.79 (C-2′), 146.77 (C-8′), 150.69 (C-8′′′a), 152.99 (C-2′′′), 158.92 (C-4′′′), 161.13 (C-6′), 173.03 (C-1) ppm. MS (EI, 70 eV): m/z (%) = 477.1/476.1/475.1 [M]^+•^ (28/39/48), 249.1/248.1/247.1 [C_13_H_14_ClN_3_]^+•^ (11/19/27), 207.1/206.1/205.1 [C_11_H_10_ClN_2_]^+^ (8/9/28), 203.1/202.1/201.1 [C_12_H_13_N_2_O]^+^ (12/18/30), 193.1/192.1/191.1 [C_10_H_8_ClN_2_]^+^ (14/11/42), 188.1/187.1 [C_11_H_11_N_2_O]^+^ (21/100), 175.1/174.1 [C_10_H_10_N_2_O]^+•^ (17/32), HRMS (ESI) calcd. [M+H]^+^ 476.18478; found 476.18466.

#### 3.2.14. 6-Methoxy-*N*-(3′-(piperazin-1′′-yl)propyl)quinolin-8-amine (**25**)

LiAlH_4_ (319.4 mg, 8.42 mmol) was added in portions to a solution of amide** 12** (439.0 mg, 1.40 mmol) in THF (dry, 30 mL) at 0°C leading to a change of color to reddish brown at once. The reaction mixture was stirred at 80°C for 3 hours; during this period the color changed to yellow. The excess of LiAlH_4_ was carefully hydrolyzed using small amounts of water; the mixture was dried (MgSO_4_) and concentrated. The residue was suspended in dichloromethane, filtered, and concentrated. Purification by flash column chromatography on basic alumina (activity level V, DCM/MeOH 20 : 1) gave compound** 25** (125.8 mg, 30%) as viscous yellow oil. IR (ATR-FTIR): $\tilde{v}$ = 3393–3261 (w, br), 2935 (w, br), 2810 (w, br), 2360 (w), 2342 (w), 1725 (w), 1651 (w), 1615 (m), 1592 (m), 1576 (m), 1518 (s), 1455 (m), 1422 (m), 1385 (m), 1320 (w), 1263 (w), 1212 (m), 1196 (w), 1169 (m), 1154 (m), 1133 (m), 1072 (w), 1051 (w), 1027 (w), 997 (w), 970 (w), 918 (w), 899 (w), 819 (m), 790 (m), 704 (w), 667 (w), 624 (w) cm^−1^. ^1^H-NMR (400 MHz, MeOD-d_4_): *δ* = 1.89–1.96 (m, 2 H, 2′-CH_2_), 2.48–2.54 (m, 6 H, 3′-CH_2_, piperazine CH_2_), 2.89 (t, ^3^ *J*_H–H_ = 4.92 Hz, 4 H, piperazine CH_2_), 3.31–3.34 (m, 2 H, 1′-CH_2_), 3.86 (s, 3 H, OMe), 6.32 (d, ^4^ *J*_H–H_ = 2.48 Hz, 1 H, 7-H), 6.45 (d, ^4^ *J*_H–H_ = 2.48 Hz, 1 H, 5-H), 7.34 (dd, ^3^ *J*_H–H_ = 4.24 Hz, 8.28 Hz, 1 H, 3-H), 8.01 (dd, ^3^ *J*_H–H_ = 8.24 Hz, ^4^ *J*_H–H_ = 1.56 Hz, 1 H, 4-H), 8.51 (dd, ^3^ *J*_H–H_ = 4.24 Hz, ^4^ *J*_H–H_ = 1.60 Hz, 1 H, 2-H) ppm. ^13^C-NMR (100 MHz, MeOD-d_4_): *δ* = 26.61 (C-2′), 43.06 (C-1′), 46.27 (piperazine CH_2_), 55.19 (piperazine CH_2_), 55.79 (OMe), 58.51 (C-3′), 93.43 (C-5), 98.22 (C-7), 123.06 (C-3), 131.58 (C-4a), 136.31 (C-4), 136.80 (C-8a), 145.58 (C-2), 147.38 (C-8), 161.17 (C-6) ppm. MS (EI, 70 eV): m/z (%) = 302.2/301.2/300.2 [M]^+•^ (3/18/60), 202.1/201.1 [C_12_H_13_N_2_O]^+^ (16/73), 188.1/187.1 [C_11_H_11_N_2_O]^+^ (19/50), 175.1/174.1 [C_10_H_10_N_2_O]^+•^ (14/22), HRMS (ESI) calcd. [M+H]^+^ 301.20229; found 301.20235.

#### 3.2.15. *N*-(3′-(4′′-(7′′′-Chloroquinolin-4′′′-yl)piperazin-1′′-yl)propyl)-6-methoxyquinolin-8-amine (**14**)

Amine** 25** (28.0 mg, 0.09 mmol) and 4,7-dichloroquinoline (19.3 mg, 0.10 mmol) were stirred at 120°C under neat conditions for 8.5 hours. The residue was suspended in methanol and alkalized by using ammonia solution, and the solvent was concentrated. Purification with flash column chromatography on deactivated silica gel (DCM/MeOH 50 : 1) gave compound** 14** (19.5 mg, 44%) as yellow oil. IR (ATR-FTIR): $\tilde{v}$ = 3393–3260 (w, br), 3046 (w, br), 2938 (w, br), 2818 (w, br), 1731 (w), 1671 (w), 1651 (w), 1608 (m), 1573 (s), 1517 (s), 1455 (m), 1422 (m), 1384 (m), 1334 (w), 1296 (w), 1264 (w), 1251 (w), 1228 (w), 1211 (m), 1194 (m), 1166 (m), 1154 (m), 1137 (m), 1070 (w), 1051 (w), 1021 (w), 993 (w), 970 (w), 949 (w), 927 (w), 898 (w), 870 (m), 819 (s), 790 (m), 770 (w), 732 (m), 700 (w), 652 (w), 629 (m) cm^−1^. ^1^H-NMR (400 MHz, MeOD-d_4_): *δ* = 2.00 (quin, ^3^ *J*_H–H_ = 6.72 Hz, 2 H, 2′-CH_2_), 2.68 (t, ^3^ *J*_H–H_ = 7.02 Hz, 2 H, 3′-CH_2_), 2.78–2.83 (m, 4 H, 2′′-CH_2_, 6′′-CH_2_), 3.33–3.40 (m, 6 H, 1′-CH_2_, 3′′-CH_2_, 5′′-CH_2_), 3.87 (s, 3 H, OMe), 6.34 (d, ^4^ *J*_H–H_ = 2.44 Hz, 1 H, 7-H), 6.46 (d, ^4^ *J*_H–H_ = 2.28 Hz, 1 H, 5-H), 7.01 (d, ^3^ *J*_H–H_ = 5.24 Hz, 1 H, 3′′′-H), 7.34 (dd, ^3^ *J*_H–H_ = 4.20 Hz, 8.28 Hz, 1 H, 3-H), 7.51 (dd, ^3^ *J*_H–H_ = 9.08 Hz, ^4^ *J*_H–H_ = 2.12 Hz, 6′′′-H), 7.93 (d, ^4^ *J*_H–H_ = 2.08 Hz, 1 H, 8′′′-H), 8.01 (dd, ^3^ *J*_H–H_ = 8.24 Hz, ^4^ *J*_H–H_ = 1.48 Hz, 1 H, 4-H), 8.05 (d, ^3^ *J*_H–H_ = 9.08 Hz, 1 H, 5′′′-H), 8.48 (dd, ^3^ *J*_H–H_ = 4.20 Hz, ^4^ *J*_H–H_ = 1.64 Hz, 1 H, 2-H), 8.64 (d, ^3^ *J*_H–H_ = 5.16 Hz, 1 H, 2′′′-H) ppm. ^13^C-NMR (100 MHz, MeOD-d_4_): *δ* = 26.82 (C-2′), 43.21 (C-1′), 53.15 (C-3′′, C-5′′), 54.35 (C-2′′, C-6′′), 55.81 (OMe), 57.97 (C-3′), 93.43 (C-5), 98.25 (C-7), 110.37 (C-3′′′), 123.09 (C-3), 123.27 (C-4′′′a), 127.34 (C-5′′′), 127.45 (C-6′′′), 128.46 (C-8′′′), 131.61 (C-4a), 136.33 (C-4), 136.65 (C-7′′′), 136.84 (C-8a), 145.57 (C-2), 147.46 (C-8), 150.77 (C-8′′′a), 153.02 (C-2′′′), 159.38 (C-4′′′), 161.19 (C-6) ppm. MS (EI, 70 eV): m/z (%) = 463.2/462.2/461.2 [M]^+•^ (19/25/30), 289.1/288.1/287.1 [C_16_H_18_ClN_3_]^+^ (18/20/49), 262.1/261.1/260.1 [C_14_H_15_ClN_3_]^+^ (14/29/29), 203.1/202.1/201.1 [C_12_H_13_N_2_O]^+^ (12/54/100), 189.1/188.1/187.1 [C_11_H_11_N_2_O]^+^ (15/37/48), 175.1/174.1 [C_10_H_10_N_2_O]^+•^ (15/21), HRMS (ESI) calcd. [M+H]^+^ 462.20551; found 462.20527.

#### 3.2.16. 3-Bromo-1-(4′-(3′′-(6′′′-methoxyquinolin-8′′′-ylamino)propanoyl)piperazin-1′-yl)propan-1-one (**26**)

A colorless solution of 3-bromopropionyl chloride** 19** (388.0 mg, 2.26 mmol, 0.02 mM) in DCM (dry, 111 mL) was slowly added over a period of time of 2 hours to a yellow suspension of aminoquinoline** 12** (704 mg, 2.24 mmol, 0.1 mM) and sodium acetate (368 mg, 4.49 mmol) in DCM (dry, 22.3 mL) at −20°C under nitrogen atmosphere. After completed addition, the orange colored reaction mixture was stirred for 1 hour at −20°C. The solvent was concentrated, the residue was repeatedly suspended in dichloromethane, and all volatile components were removed under reduced pressure. The residue was suspended in dichloromethane, filtered (Celite), and concentrated. Purification on silica gel (ethyl acetate 100%) gave compound** 26** (690.8 mg, 1.537, 69%) as light yellow solid. Mp 98°C (ethyl acetate), IR (ATR-FTIR): $\tilde{v}=3399$ (w, br), 2922 (w, br), 1737 (w), 1615 (s), 1517 (m), 1426 (s), 1387 (m), 1282 (m), 1213 (s), 1166 (m), 1014 (m), 821 (m), 791 (m), 621 (m), 610 (m) cm^−1^. ^1^H-NMR (600 MHz, MeOD-d_4_): *δ* = 2.80 (t, ^3^ *J*_H–H_ = 6.22 Hz, 2 H, 2′′-CH_2_), 2.91 (t, ^3^ *J*_H–H_ = 6.66 Hz, 1 H, 2-CH_2_), 2.97 (t, ^3^ *J*_H–H_ = 6.66 Hz, 1 H, 2-CH_2_), 3.39-3.40 (m, 1 H, piperazine CH_2_), 3.46–3.54 (m, 5 H, piperazine CH_2_), 3.57–3.65 (m, 6 H, 3′′-CH_2_, 3-CH_2_, piperazine CH_2_), 3.87 (s, 3 H, OMe), 6.39 (s, 1 H, 7′′′-H), 6.48 (d, ^4^ *J*_H–H_ = 2.04 Hz, 5′′′-H), 7.34 (dd, ^3^ *J*_H–H_ = 3.96 Hz, 8.16 Hz, 1 H, 3′′′-H), 8.02 (d, ^3^ *J*_H–H_ = 8.16 Hz, 1 H, 4′′′-H), 8.49 (m, 1 H, 2′′′-H) ppm. ^13^C-NMR (150 MHz, MeOD-d_4_): *δ* = 28.00 (C-3), 33.28 (C-2′′), 33.36 (C-2′′), 37.02 (C-2), 37.06 (C-2), 40.36 (C-3′′), 40.40 (C-3′′), 42.57 (piperazine CH_2_), 42.58 (piperazine CH_2_), 42.89 (piperazine CH_2_), 42.90 (piperazine CH_2_), 46.24 (piperazine CH_2_), 46.48 (piperazine CH_2_), 46.72 (piperazine CH_2_), 55.84 (OMe), 55.86 (OMe), 93.81 (C-5′′′), 98.39 (C-7′′′), 98.40 (C-7′′′), 123.19 (C-3′′′), 123.21 (C-3′′′), 131.57 (C-4′′′a), 131.63 (C-4′′′a), 136.32 (C-4′′′), 136.38 (C-4′′′), 136.65 (C-8′′′a), 136.66 (C-8′′′a), 145.74 (C-2′′′), 145.76 (C-2′′′), 161.03 (C-6′′′), 161.08 (C-6′′′), 171.26 (C-1), 171.30 (C-1), 172.99 (C-1′′), 173.04 (C-1′′) ppm. MS (EI, 70 eV): m/z (%) = 371.2/370.2/369.2 [M-Br]^+^ (8/20/33), 203.1/202.1/201.1 [C_12_H_13_N_2_O]^+^ (25/48/44), 188.1/187.1 [C_11_H_11_N_2_O]^+^ (71/92), 175.1/174.1 [C_10_H_10_N_2_O]^+•^ (17/19), 81.9/79.9 [HBr]^+^ (99/100), 80.9/78.9 [Br]^+^ (44/37), HRMS (ESI) calcd. [M]^+•^ 448.11045; found 448.11047.

#### 3.2.17. 1-(4′-(3′′-(6′′′-Methoxyquinolin-8′′′-ylamino)propanoyl)piperazin-1′-yl)prop-2-en-1-one (Not Displayed)

During the synthesis of compound** 26**, a side product was obtained and synthesized for analysis and biotests. Cs_2_CO_3_ (128.9 mg, 0.40 mmol) was added to a solution of the bromine derivative** 26** (118.7 mg, 0.26 mmol) in DCM (dry, 15 mL) and the reaction mixture was stirred at 25°C overnight. The suspension was filtered (MgSO_4_) and concentrated, and the residue was purified with flash column chromatography on deactivated silica gel (DCM/MeOH 10 : 1) to give the side product (86.5 mg, 0.23 mmol, 90%) as light yellow solid. Mp 153°C (DCM/MeOH), IR (ATR-FTIR): $\tilde{v}=3384$ (w, br), 2861 (w, br), 2360 (w), 2341 (w), 1638 (s), 1611 (s), 1576 (m), 1517 (m), 1425 (s), 1386 (s), 1276 (w), 1212 (s), 1197 (s), 1165 (m), 1154 (m), 1125 (w), 1050 (w), 1018 (m), 975 (m), 906 (w), 820 (m), 789 (s), 668 (w), 632 (w), 620 (w), 604 (w) cm^−1^. ^1^H-NMR (400 MHz, MeOD-d_4_): *δ* = 2.82 (t, ^3^ *J*_H–H_ = 6.24 Hz, 2 H, 2′′-CH_2_), 3.55–3.67 (m, 10 H, piperazine CH_2_, 3′′-CH_2_), 3.87 (s, 3 H, OMe), 5.73 (d, ^3^ *J*_H–H_ = 8.92 Hz, 1 H, 3-CH_2_), 6.18 (d, ^3^ *J*_H–H_ = 16.96 Hz, 1 H, 3-CH_2_), 6.39 (s, 1 H, 7′′′-H), 6.48 (d, ^4^ *J*_H–H_ = 2.40 Hz, 1 H, 5′′′-H), 6.62–6.75 (m, 1 H, 2-H), 7.35 (dd, ^3^ *J*_H–H_ = 4.24 Hz, 8.24 Hz, 1 H, 3′′′-H), 8.01 (d, ^3^ *J*_H–H_ = 8.28 Hz, 1 H, 4′′′-H), 8.49 (dd, ^3^ *J*_H–H_ = 4.16 Hz, ^4^ *J*_H–H_ = 1.60 Hz, 1 H, 2′′′-H) ppm. ^13^C-NMR (100 MHz, MeOD-d_4_): *δ* = 33.32 (C-2′′), 40.40 (C-3′′), 43.04 (b, piperazine CH_2_), 43.13 (b, piperazine CH_2_), 46.51 (b, piperazine CH_2_), 46.74 (b, piperazine CH_2_), 55.84 (OMe), 93.82 (C-5′′′), 98.35 (C-7′′′), 123.18 (C-3′′′), 128.82 (C-2), 129.01 (C-3), 131.63 (C-4′′′a), 136.31 (C-4′′′), 136.73 (C-8′′′a), 145.77 (C-2′′′), 146.79 (C-8′′′), 161.09 (C-6′′′), 167.80 (C-1), 173.02 (C-1′′) ppm. MS (EI, 70 eV): m/z (%) = 370.2/369.2/368.2 [M]^+•^ (8/31/58), 202.1/201.1 [C_12_H_13_N_2_O]^+^ (25/59), 188.1/187.1 [C_11_H_11_N_2_O]^+^ (38/100), 175.1/174.1 [C_10_H_10_N_2_O]^+•^ (10/18), 160.1/159.1 [C_10_H_9_NO]^+•^ (7/14), HRMS (ESI) calcd. [M]^+•^ 368.18429; found 368.18412.

#### 3.2.18. 3-Azido-1-(4′-(3′′-(6′′′-methoxyquinolin-8′′′-ylamino)propanoyl)piperazin-1-yl)propan-1-one (**27**)

A suspension consisting of the bromine derivative** 26** (574.5 mg, 1.28 mmol) and NaN_3_ (250.2 mg, 3.85 mmol) in DMF (dry, 25 mL) was stirred under nitrogen atmosphere overnight at 25°C. The complete conversion was determined by ^1^H-NMR spectroscopy. An aqueous solution of NaHCO_3_ was added and exhaustively extracted with dichloromethane. The combined organic extracts were dried (MgSO_4_), filtered, and concentrated. Purification with column chromatography on deactivated silica gel (ethyl acetate 100%) gave** 27** (486.8 mg, 1.18 mmol, 92%) as light yellow solid. Mp 86°C (ethyl acetate), IR (ATR-FTIR): $\tilde{v}=3385$ (w), 2933–2860 (w, br), 2504 (w), 2101 (m), 1633 (s), 1576 (m), 1518 (m), 1501 (m), 1423 (s), 1386 (s), 1282 (m), 1211 (s), 1154 (m), 1126 (m), 1051 (m), 1016 (m), 901 (w), 821 (m), 790 (m), 665 (w), 625 (m) cm^−1^. ^1^H-NMR (600 MHz, MeOD-d_4_): *δ* = 2.58 (t, ^3^ *J*_H–H_ = 6.24 Hz, 1 H, 2-CH_2_), 2.64 (t, ^3^ *J*_H–H_ = 6.24 Hz, 1 H, 2-CH_2_), 2.82 (t, ^3^ *J*_H–H_ = 6.30 Hz, 2 H, 2′′-CH_2_), 3.40–3.43 (m, 1 H, piperazine CH_2_), 3.48–3.60 (m, 8 H, piperazine CH_2_, 3-CH_2_), 3.64-3.65 (m, 3 H, piperazine CH_2_, 3′′-CH_2_), 3.87 (s, 3 H, OMe), 6.39 (d, ^4^ *J*_H–H_ = 2.28 Hz, 1 H, 5′′′-H), 6.49 (d, ^4^ *J*_H–H_ = 2.34 Hz, 1 H, 7′′′-H), 7.35 (dd, ^3^ *J*_H–H_ = 4.14 Hz, 8.16 Hz, 1 H, 3′′′-H), 8.02 (d, ^3^ *J*_H–H_ = 8.28 Hz, 1 H, 4′′′-H), 8.50 (s, br, 1 H, 2′′′-H) ppm. ^13^C-NMR (150 MHz, MeOD-d_4_): *δ* = 33.30 (CH_2_), 33.33 (CH_2_), 33.37 (CH_2_), 40.37 (C-3′′), 42.53 (piperazine CH_2_), 42.58 (piperazine CH_2_), 42.86 (piperazine CH_2_), 42.90 (piperazine CH_2_), 46.22 (piperazine CH_2_), 46.44 (piperazine CH_2_), 46.48 (piperazine CH_2_), 46.74 (piperazine CH_2_), 48.37 (C-1), 55.83 (OMe), 93.80 (C-5′′′), 98.36 (C-7′′′), 123.20 (C-3′′′), 131.64 (C-4′′′a), 136.33 (C-4′′′), 136.74 (C-8′′′a), 145.78 (C-2′′′), 146.78 (C-8′′′), 161.10 (C-6′′′), 171.51 (C-1), 171.57 (C-1), 173.03 (C-1′′), 173.09 (C-1′′) ppm. MS (EI, 70 eV): m/z (%) = 413.2/412.2/411.1 [M]^+•^ (3/8/13), 385.2/384.1/383.1 [M-N_2_]^+•^ (5/14/17), 370.1/369.1/368.1 [M-HN_3_]^+•^ (3/4/4), 203.0/202.1/201.1 [C_12_H_13_N_2_O]^+^ (7/26/43), 188.1/187.0 [C_11_H_11_N_2_O]^+^ (21/100), 175.0/174.0 [C_10_H_10_N_2_O]^+•^ (13/37), HRMS (ESI) calcd. [M]^+•^ 411.20134; found 411.20129.

#### 3.2.19. 3-Amino-1-(4′-(3′′-(6′′′-methoxyquinolin-8′′′-ylamino)propanoyl)piperazin-1-yl)propan-1-one (**28**)

A solution of azide** 27** (57.9 mg, 0.14 mmol) and PPh_3_ (47.2 mg, 0.18 mmol) in MeOH (dry, 5 mL) was stirred at 25°C under nitrogen atmosphere overnight. The solvent was concentrated and the residue was purified with flash column chromatography on alumina (activity level V, gradient elution starting with ethyl acetate 100%, followed by DCM/MeOH 1 : 2). Compound** 28** (43.9 mg, 0.11 mmol, 81%) was obtained as light yellow solid. Mp 65°C (DCM/MeOH), IR (ATR-FTIR): $\tilde{v}=3378$ (w, br), 2922 (w), 2854 (w), 2360 (m), 2341 (w), 1734 (w), 1615 (s), 1576 (m), 1517 (s), 1423 (s), 1385 (s), 1336 (w), 1282 (w), 1213 (m), 1196 (s), 1165 (m), 1154 (m), 1124 (w), 1048 (w), 1019 (m), 985 (w), 899 (w), 821 (m), 790 (m), 722 (w), 668 (w), 624 (w), 613 (w) cm^−1^. ^1^H-NMR (600 MHz, MeOD-d_4_): *δ* = 2.50 (t, ^3^ *J*_H–H_ = 6.28 Hz, 1 H, CH_2_), 2.55 (t, ^3^ *J*_H–H_ = 6.36 Hz, 1 H, CH_2_), 2.81 (t, ^3^ *J*_H–H_ = 6.36 Hz, 2 H, 2′′-CH_2_), 2.88–2.92 (m, 2 H, CH_2_), 3.40-3.41 (m, 1 H, piperazine CH_2_), 3.46–3.55 (m, 5 H, piperazine CH_2_), 3.57–3.59 (m, 1 H, piperazine CH_2_), 3.62–3.65 (m, 3 H, piperazine CH_2_), 3.87 (s, 3 H, OMe), 6.39 (d, ^4^ *J*_H–H_ = 2.40 Hz, 1 H, 7′′′-H), 6.48 (d, ^4^ *J*_H–H_ = 2.22 Hz, 5′′′-H), 7.34–7.36 (m, 1 H, 3′′′-H), 8.02 (d, br, ^3^ *J*_H–H_ = 8.28 Hz, 4′′′-H), 8.49 (m, br, 2′′′-H) ppm. ^13^C-NMR (150 MHz, MeOD-d_4_): *δ* = 33.30 (C-2′′), 33.37 (C-2′′), 35.70 (CH_2_), 35.75 (CH_2_), 38.35 (CH_2_), 40.40 (C-3′′), 42.34 (piperazine CH_2_), 42.59 (piperazine CH_2_), 42.67 (piperazine CH_2_), 42.88 (piperazine CH_2_), 46.12 (piperazine CH_2_), 46.35 (piperazine CH_2_), 46.48 (piperazine CH_2_), 46.70 (piperazine CH_2_), 55.84 (OMe), 93.78 (C-5′′′), 98.34 (C-7′′′), 123.18 (C-3′′′), 131.63 (C-4′′′a), 136.32 (C-4′′′), 136.72 (C-8′′′a), 145.76 (C-2′′′), 146.78 (C-8′′′), 161.08 (C-6′′′), 172.48 (C-1), 172.52 (C-1), 172.98 (C-1′′), 173.05 (C-1′′) ppm. MS (EI, 70 eV): m/z (%) = 387.2/386.2/385.2 [M]^+•^ (3/10/14), 370.2/369.2/368.2 [C_20_H_24_N_4_O_3_]^+•^ (4/13/19), 203.1/202.1/201.1 [C_12_H_13_N_2_O]^+^ (5/24/42), 188.1/187.1 [C_11_H_11_N_2_O]^+^ (17/100), 175.1/174.1 [C_10_H_10_N_2_O]^+•^ (11/30), HRMS (ESI) calcd. [M+H]^+^ 386.21867; found 386.21859.

#### 3.2.20. 3-(7′′′′-Chloroquinolin-4′′′′-ylamino)-1-(4′-(3′′-(6′′′-methoxyquinolin-8′′′-ylamino)propanoyl)piperazin-1′-yl)propan-1-one (**15**)

Amine** 28** (31.3 mg, 0.08 mmol) and 4,7-dichloroquinoline (12.4 mg, 0.06 mmol) were stirred at 100°C under neat conditions for 8 hours. The residue was suspended in a mixture of dichloromethane and methanol, the orange colored suspension was alkalized using aqueous ammonia solution leading to a change of color to yellow, and all volatile components were removed under reduced pressure. The residue was purified by preparative thin layer chromatography on deactivated silica gel (repeated elution with DCM/MeOH 20 : 1). Compound** 15** (9.8 mg, 0.02 mmol, 30%) was obtained as beige solid. Mp 131°C (DCM/MeOH), IR (ATR-FTIR): $\tilde{v}=3393$ (w), 3235 (w), 3059 (w), 2918 (w), 1736 (w), 1644 (m), 1615 (m), 1573 (s), 1519 (m), 1425 (m), 1387 (m), 1366 (m), 1329 (w), 1283 (w), 1219 (m), 1166 (m), 1133 (m), 1078 (w), 1049 (w), 1023 (m), 995 (m), 921 (w), 876 (w), 844 (w), 812 (m), 788 (m), 732 (w), 681 (w), 644 (w), 620 (w) cm^−1^. ^1^H-NMR (600 MHz, CD_2_Cl_2_): *δ* = 2.68–2.74 (m, 4 H, 2-CH_2_), 3.39–3.43 (m, 4 H, piperazine CH_2_), 3.62–3.67 (m, 8 H, piperazine CH_2_, 3-CH_2_, 3′′-CH_2_), 3.87 (s, 3 H, OMe), 6.13 (s, 1 H, NH), 6.32 (d, ^3^ *J*_H–H_ = 2.22 Hz, 1 H, 7′′′-H), 6.38 (s, 1 H, 5′′′-H), 6.42–6.48 (m, 3′′′′-H, NH), 7.29-7.39 (m, 2 H, 3′′′-H, 5′′′′-H), 7.71-7.72 (m, 1 H, 6′′′′-H), 7.90 (s, 1 H, 8′′′′-H), 7.94 (d, ^3^ *J*_H–H_ = 8.16 Hz, 1 H, 4′′′-H), 8.48–8.52 (m, 2 H, 2′′′-H, 2′′′′-H) ppm. ^13^C-NMR (150 MHz, CD_2_Cl_2_): *δ* = 31.87 (C-2/2′′), 31.90 (C-2/2′′), 32.63 (C-2/2′′), 32.71 (C-2/2′′), 39.04 (C-3/3′′), 39.08 (C-3/3′′), 39.30 (C-3/3′′), 39.34 (C-3/3′′), 41.55 (piperazine CH_2_), 41.65 (piperazine CH_2_), 41.68 (piperazine CH_2_), 41.77 (piperazine CH_2_), 45.43 (piperazine CH_2_), 45.49 (piperazine CH_2_), 45.56 (piperazine CH_2_), 55.72 (OMe), 92.59 (C-5′′′), 96.94 (C-7′′′), 99.22 (C-3′′′′), 118.00 (C-4′′′′a), 122.14 (C-6′′′′), 122.48 (C-3′′′), 125.64 (C-5′′′′), 128.90 (C-8′′′′), 130.36 (C-4′′′a), 135.19 (C-4′′′), 135.27 (C-7′′′′), 135.84 (C-8′′′a), 145.03 (C-2′′′), 145.92 (C-8′′′), 149.62 (C-8′′′′a), 150.16 (C-4′′′′), 152.35 (C-2′′′′), 159.94 (C-6′′′), 170.40 (C-1/1′′), 170.46 (C-1/1′′), 170.56 (C-1/1′′), 170.62 (C-1/1′′) ppm. MS (EI, 70 eV): m/z (%) = 549.1/548.1/547.1/546.1 [M]^+•^ (3/9/9/22), 375.1/374.1/373.1/372.1 [C_19_H_22_ClN_4_O_2_]^+^ (3/5/8/9), 320.1/319.1/318.1 [C_16_H_19_ClN_4_O]^+•^ (12/15/28), 316.1/315.1/314.1 [C_17_H_22_N_4_O_2_]^+•^ (2/4/5), 207.0/206.0/205.0 [C_11_H_10_ClN_2_]^+^ (33/22/100), 203.0/202.1/201.1 [C_12_H_13_N_2_O]^+^ (7/8/41), 193.0/192.0/191.0 [C_10_H_8_ClN_2_]^+^ (10/9/29), 188.1/187.1 [C_11_H_11_N_2_O]^+^ (13/79), 180.0/179.0/178.0 [C_9_H_7_ClN_2_]^+•^ (17/20/46), 175.1/174.1 [C_10_H_10_N_2_O]^+•^ (13/56), HRMS (ESI) calcd. [M+H]^+^ 547.22189; found 547.22165.

#### 3.2.21. 3-((7′′′′′-Chloroquinolin-4′′′′′-yl)(6′′′′-methoxyquinolin-8′′′′-yl)amino)-1-(4′-(3′′-(7′′′-chloroquinolin-4′′′-ylamino)propanoyl)piperazin-1′-yl)propan-1-one (**17**)

Amine** 28** (26.1 mg, 0.07 mmol) and 4,7-dichloroquinoline (41.8 mg, 0.21 mmol) were stirred at 120°C under neat conditions for 8.5 hours. The residue was suspended in a solvent mixture containing dichloromethane and methanol; the dark red suspension was alkalized using aqueous ammonia solution leading to a change of color to light brownish color and all volatile components were removed under reduced pressure. The residue was purified by preparative thin layer chromatography (repeated elution with DCM/MeOH 25 : 1) to give product** 17** (14.0 mg, 0.02 mmol, 28%) as yellow solid. Mp 152–155°C (DCM/MeOH), IR (ATR-FTIR): $\tilde{v}=3374$ (w, br), 2936 (w, br), 2360 (s), 2341 (m), 1607 (s), 1575 (s), 1522 (m), 1421 (m), 1368 (m), 1277 (w), 1211 (m), 1135 (w), 1108 (w), 1074 (w), 1016 (m), 915 (w), 877 (m), 808 (m), 784 (m), 763 (m), 680 (w), 669 (w), 643 (w), 630 (w), 609 (w) cm^−1^. ^1^H-NMR (600 MHz, MeOD-d_4_): *δ* = 2.74 (t, ^3^ *J*_H–H_ = 6.50 Hz, 2 H, 2′′-CH_2_), 2.82 (t, ^3^ *J*_H–H_ = 6.66 Hz, 2 H, 2′′-CH_2_), 2.85–2.91 (m, 4 H, 2-CH_2_), 3.40–3.42 (m, 2 H, piperazine CH_2_), 3.46–3.48 (m, 2 H, piperazine CH_2_), 3.52–3.54 (m, 4 H, piperazine CH_2_), 3.56–3.61 (m, 8 H, piperazine CH_2_), 3.65–3.70 (m, 4 H, 3′′-CH_2_), 3.78–3.82 (m, 10 H, OMe, 3-CH_2_), 6.55 (d, ^3^ *J*_H–H_ = 5.82 Hz, 1 H, 3′′′-H), 6.60 (d, ^3^ *J*_H–H_ = 5.88 Hz, 1 H, 3′′′-H), 6.75 (s, 2 H, 7′′′′-H), 7.12 (dd, ^3^ *J*_H–H_ = 4.08 Hz, 8.58 Hz, 1 H, 3′′′′-H), 7.17 (dd, ^3^ *J*_H–H_ = 4.02 Hz, 8.58 Hz, 1 H, 3′′′′-H), 7.27 (d, ^3^ *J*_H–H_ = 7.62 Hz, 1 H, 4′′′′-H), 7.31 (d, ^3^ *J*_H–H_ = 7.32 Hz, 1 H, 4′′′′-H), 7.36–7.41 (m, 8 H, 6′′′-H, 3′′′′′-H, 5′′′′-H, 6′′′′-H), 7.77–7.79 (m, 2 H, 8′′′-H), 8.01 (d, ^3^ *J*_H–H_ = 9.00 Hz, 1 H, 5′′′-H), 8.04 (d, ^3^ *J*_H–H_ = 9.00 Hz, 1 H, 5′′′-H), 8.34 (d, ^3^ *J*_H–H_ = 5.88 Hz, 1 H, 2′′′-H), 8.36 (d, ^3^ *J*_H–H_ = 5.88 Hz, 1 H, 2′′′-H), 8.49-8.50 (m, 2 H, 2′′′′-H), 8.92 (d, ^3^ *J*_H–H_ = 4.44 Hz, 2 H, 2′′′′′-H) ppm. 5′-H was not detectable because of the proton-deuterium exchange at this position. ^13^C-NMR (150 MHz, MeOD-d_4_): *δ* = 32.62 (C-2′′), 32.65 (C-2′′), 33.48 (C-2), 33.81 (C-2), 40.06 (C-3′′), 40.17 (C-3′′), 40.30 (C-3), 40.39 (C-3), 42.45 (piperazine CH_2_), 42.62 (piperazine CH_2_), 42.82 (piperazine CH_2_), 42.89 (piperazine CH_2_), 46.28 (piperazine CH_2_), 46.42 (piperazine CH_2_), 46.52 (piperazine CH_2_), 46.74 (piperazine CH_2_), 56.76 (OMe), 92.92 (C-7′′′′), 99.76 (C-3′′′), 99.82 (C-3′′′), 105.88 (C-5′′′′), 105.92 (C-5′′′′), 118.62 (C-4′′′a), 123.79 (C-3′′′′), 124.46 (C-5′′′), 124.49 (C-5′′′), 126.42 (C-3′′′′′), 126.44 (C-3′′′′′), 126.64 (C-6′′′), 126.67 (C-6′′′), 126.82 (C-8′′′), 126.88 (C-8′′′), 128.47 (C-8′′′′′), 128.70 (C-6′′′′′), 128.72 (C-6′′′′′), 129.17 (C-4′′′′′a), 129.56 (C-5′′′′′), 129.60 (C-5′′′′′), 129.95 (C-4′′′′a), 133.37 (C-4′′′′), 133.42 (C-4′′′′), 135.14 (C-8′′′′a), 135.21 (C-8′′′′a), 136.80 (C-7′′′′′), 137.17 (C-7′′′), 137.20 (C-7′′′), 145.96 (C-2′′′′), 146.85 (C-4′′′′′), 146.88 (C-4′′′′′), 148.43 (C-8′′′′), 148.55 (C-8′′′a), 148.60 (C-8′′′a), 149.75 (C-8′′′′′a), 151.47 (C-2′′′), 151.53 (C-2′′′), 152.35 (C-2′′′′′), 153.07 (C-4′′′), 153.19 (C-4′′′), 157.74 (C-6′′′′), 172.15 (C-1′′), 172.21 (C-1′′), 173.05 (C-1), 173.09 (C-1) ppm. MS (EI, 70 eV): m/z (%) = 338.1/337.1/336.1/335.1 [C_19_H_14_ClN_3_O]^+•^ (11/37/34/100), 323.1/322.1/321.1 [C_18_H_12_ClN_3_O]^+•^ (14/16/41), 207.1/206.1/205.1 [C_11_H_10_ClN_2_]^+^ (11/9/28), 192.1/191.1 [C_10_H_8_ClN_2_]^+^ (10/11), 180.1/179.1/178.1 [C_9_H_7_ClN_2_]^+•^ (10/9/29), HRMS (ESI) calcd. [M+H]^+^ 708.22512; found 708.22501.

#### 3.2.22. *N*-(3′-(4′′-(3′′′-Aminopropyl)piperazin-1′′-yl)propyl)-6-methoxyquinolin-8-amine (**29**)

LiAlH_4_ (82.9 mg, 2.18 mmol) was added to a solution of diamide** 27** (149.7 mg, 0.36 mmol) in THF (dry, 20 mL) leading to a change of color to orange at once. The reaction mixture was stirred at 80°C for 3 hours, and the excess of LiAlH_4_ was carefully hydrolyzed using water leading to decolorization. The reaction mixture was dried (Na_2_SO_4_), filtered, and concentrated. The residue was suspended in dichloromethane, filtered, and concentrated. Purification by flash column chromatography on alumina (activity level V, gradient elution with ethyl acetate 100%, followed by DCM/MeOH 10 : 1) gave compound** 29** (44.0 mg, 0.12 mmol, 34%) as yellow oil. IR (ATR-FTIR): $\tilde{v}=3388$ (w, br), 2937 (w), 2876 (w), 2813 (w), 1615 (m), 1592 (m), 1576 (m), 1518 (s), 1456 (m), 1422 (m), 1385 (m), 1350 (w), 1334 (w), 1308 (w), 1267 (w), 1237 (w), 1212 (m), 1196 (m), 1154 (s), 1051 (w), 1027 (w), 994 (w), 969 (w), 943 (w), 919 (w), 899 (w), 818 (s), 789 (m), 764 (w), 751 (w), 667 (w), 625 (w) cm^−1^. ^1^H-NMR (600 MHz, MeOD-d_4_): *δ* = 1.65–1.70 (m, 2 H, 2′′′-CH_2_), 1.89-1.90 (m, 2 H, 2′-CH_2_), 2.41–2.71 (m, 14 H, 3′-CH_2_, piperazine CH_2_, 1′′′-CH_2_, 3′′′-CH_2_), 3.30–3.32 (m, 2 H, 1′-CH_2_), 3.86 (s, 3 H, OMe), 6.31 (d, ^4^ *J*_H–H_ = 2.46 Hz, 1 H, 7-H), 6.45 (d, ^4^ *J*_H–H_ = 2.40 Hz, 1 H, 5-H), 7.34 (dd, ^3^ *J*_H–H_ = 4.20 Hz, 8.28 Hz, 1 H, 3-H), 8.01 (dd, ^3^ *J*_H–H_ = 8.28 Hz, ^4^ *J*_H–H_ = 1.44 Hz, 1 H, 4-H), 8.50 (dd, ^3^ *J*_H–H_ = 4.70 Hz, ^4^ *J*_H–H_ = 1.62 Hz, 1 H, 2-H) ppm. ^13^C-NMR (150 MHz, MeOD-d_4_): *δ* = 26.84 (C-2′), 30.02 (C-2′′′), 41.09 (C-3′′′), 42.98 (C-1′), 53.98 (piperazine CH_2_), 54.10 (piperazine CH_2_), 55.80 (OMe), 57.49 (C-1′′′), 57.81 (C-3′), 93.40 (C-5), 98.22 (C-7), 123.08 (C-3), 131.58 (C-4a), 136.33 (C-4), 136.76 (C-8a), 145.58 (C-2), 147.33 (C-8), 161.15 (C-6) ppm. MS (EI, 70 eV): m/z (%) = 358.2/357.2 [M]^+•^ (12/40), 245.1/244.1 [C_14_H_18_N_3_O]^+^ (7/38), 202.1/201.1 [C_12_H_13_N_2_O]^+^ (15/100), 188.1/187.1 [C_11_H_11_N_2_O]^+^ (13/39), 175.1/174.1 [C_10_H_10_N_2_O]^+•^ (11/23), HRMS (ESI) calcd. [M+H]^+^ 358.26014; found 358.26021.

#### 3.2.23. 7-Chloro-*N*-(3′-(4′′-(3′′′-(6′′′′-methoxyquinolin-8′′′′-ylamino)propyl)piperazin-1-yl)propyl)quinolin-4-amine (**16**)

Amine** 29** (32.1 mg, 0.090 mmol) and 4,7-dichloroquinoline (13.7 mg, 0.069 mmol) were stirred at 120°C under neat conditions for 6.5 hours. The reaction mixture was suspended in methanol and alkalized using aqueous ammonia solution, and all volatile components were removed under reduced pressure. The residue was suspended in a solvent mixture of DCM/MeOH 10 : 1, filtered (Celite), and concentrated. Purification by preparative thin layer chromatography on deactivated silica gel (DCM/MeOH 20 : 1) gave** 16** (6.2 mg, 0.01 mmol, 17%) as beige powder. Mp 52°C (DCM/MeOH), IR (ATR-FTIR): $\tilde{v}=3250$ (w, br), 2922 (m), 2851 (w), 2819 (w), 1738 (w), 1612 (m), 1578 (s), 1519 (m), 1451 (m), 1423 (w), 1385 (m), 1331 (w), 1309 (w), 1280 (w), 1212 (w), 1153 (m), 1138 (m), 1077 (w), 1051 (w), 1029 (w), 989 (w), 900 (w), 872 (w), 852 (w), 817 (m), 789 (m), 764 (m), 646 (w), 624 (w) cm^−1^. ^1^H-NMR (600 MHz, CD_2_Cl_2_): *δ* = 1.96–2.00 (m, 4 H, 2′-CH_2_, 2′′′-CH_2_), 2.50–2.95 (m, 12 H, 3′-CH_2_, 1′′′-CH_2_, piperazine CH_2_), 3.34–3.44 (m, 4 H, 1′-CH_2_, 3′′′-CH_2_), 3.88 (s, 3 H, OMe), 6.29 (d, ^4^ *J*_H–H_ = 2.22 Hz, 1 H, 7′′′′-H), 6.35–6.37 (m, 2 H, 3-H, 5′′′′-H), 6.81 (s, br, 1 H, NH), 7.32 (dd, ^3^ *J*_H–H_ = 4.14 Hz, 8.22 Hz, 1 H, 3′′′′-H), 7.38 (dd, ^3^ *J*_H–H_ = 8.94 Hz, ^4^ *J*_H–H_ = 2.04 Hz, 1 H, 5-H), 7.94-8.04 (m, 4 H, 6-H, 8-H, 4′′′′-H, NH), 8.45 (d, ^3^ *J*_H–H_ = 5.46 Hz, 1 H, 2-H), 8.53 (dd, ^3^ *J*_H–H_ = 4.14 Hz, ^4^ *J*_H–H_ = 1.56 Hz, 1 H, 2′′′′-H) ppm. ^13^C-NMR (150 MHz, CD_2_Cl_2_): *δ* = 23.66 (C-2′), 26.28 (C-2′′′), 42.92 (C-3′′′), 44.95 (C-1′), 53.64 (piperazine CH_2_), 53.73 (piperazine CH_2_), 53.90 (piperazine CH_2_), 54.10 (piperazine CH_2_), 55.68 (OMe), 57.63 (C-1′′′), 59.16 (C-3′), 92.12 (C-5′′′′), 96.75 (C-7′′′′), 98.82 (C-3), 117.88 (C-4a), 122.35 (C-3′′′′), 123.61 (C-8), 125.28 (C-5), 128.12 (C-6), 130.31 (C-4′′′′a), 135.11 (C-4′′′′), 135.38 (C-7), 135.96 (C-8′′′′a), 144.80 (C-2′′′′), 146.87 (C-8′′′′), 148.73 (C-8a), 151.66 (C-2, C-4), 160.12 (C-6′′′′) ppm. MS (EI, 70 eV): m/z (%) = 520.1/519.1/518.1 [M]^+•^ (15/17/36), 327.1 [M-C_10_H_8_ClN_2_]^+^ (19), 250.1/249.1/248.1 [C_13_H_15_ClN_3_]^+^ (34/19/100), 231.1 [C_13_H_17_N_3_O]^+•^ (9), 202.1/201.1 [C_12_H_13_N_2_O]^+^ (11/57), 194.0/193.0/192.0/191.0 [C_10_H_8_ClN_2_]^+^ (20/18/59/32), 188.1/187.1 [C_11_H_11_N_2_O]^+^ (15/49), 179.0/178.0 [C_9_H_7_ClN_2_]^+^ (11/7), 175.1/174.1 [C_10_H_10_N_2_O]^+•^ (9/15), 160.1/159.1 [C_10_H_9_NO]^+•^ (7/18), HRMS (ESI) calcd. [M+H]^+^ 519.26336; found 519.26361.

#### 3.2.24. 7-Chloro-*N*-(3′′-(4′′′-(3′′′′-(7′′′′′-chloroquinolin-4′′′′′-ylamino)propyl)piperazin-1′′′-yl)propyl)-*N*-(6′-methoxyquinolin-8′-yl)quinolin-4-amine (**18**)

Amine** 29** (57.9 mg, 0.16 mmol) and 4,7-dichloroquinoline (161.1 mg, 0.81 mmol) were stirred at 125°C under neat conditions for 9 hours. The residue was suspended in methanol and alkalized using aqueous ammonia solution, and all volatile components were removed under reduced pressure. The residue was suspended in a solvent mixture containing DCM/MeOH 10 : 1 and filtered over deactivated silica gel. Preparative thin layer chromatography on deactivated silica gel (DCM/MeOH 20 : 1) gave compound** 18** (25.4 mg, 0.037 mmol, 23%) as yellow powder. Mp 89°C (DCM/MeOH), IR (ATR-FTIR): $\tilde{v}=3247$ (w, br), 2928 (w), 2815 (w), 1737 (w), 1607 (m), 1576 (s), 1523 (m), 1449 (w), 1371 (m), 1331 (w), 1282 (w), 1235 (w), 1137 (m), 1109 (w), 1075 (w), 1030 (w), 988 (w), 916 (w), 875 (m), 849 (w), 808 (m), 783 (w), 734 (w), 680 (w), 637 (w), 620 (w), 611 (w) cm^−1^. ^1^H-NMR (400 MHz, CD_2_Cl_2_): *δ* = 1.97–2.10 (m, 4 H, 2′′-CH_2_, 2′′′′-CH_2_), 2.70–2.89 (m, 12 H, 3′′-CH_2_, 1′′′′-CH_2_, piperazine CH_2_), 3.38–3.47 (m, 2 H, 3′′′′-CH_2_), 3.51–3.56 (m, 2 H, 1′′-CH_2_), 3.79 (s, 3 H, OMe), 6.36 (d, ^3^ *J*_H–H_ = 5.36 Hz, 1 H, 3′′′′′-H), 6.56 (s, 1 H, 7′-H), 7.17 (dd, ^3^ *J*_H–H_ = 4.04 Hz, 8.52 Hz, 1 H, 3′-H), 7.30–7.42 (m, 5 H, 3-H, 5-H, 6-H, 4′-H, 6′′′′′-H), 7.75 (s, br, NH), 7.91 (s, 1 H, 8′′′′′-H), 7.99–8.01 (m, 1 H, 5′′′′′-H), 8.15 (d, ^4^ *J*_H–H_ = 2.04 Hz, 1 H, 8-H), 8.46 (d, ^3^ *J*_H–H_ = 5.36 Hz, 1 H, 2′′′′′-H), 8.56 (dd, ^3^ *J*_H–H_ = 4.04 Hz, ^4^ *J*_H–H_ = 1.56 Hz, 1 H, 2′-H), 8.97 (d, ^3^ *J*_H–H_ = 4.36 Hz, 1 H, 2-H) ppm. 5′-H was not detectable because of the proton-deuterium exchange at this position. ^13^C-NMR (100 MHz, CD_2_Cl_2_): *δ* = 24.02 (C-2′′′′), 26.33 (C-2′′), 43.19 (C-1′′), 45.19 (C-3′′′′), 53.94 (piperazine CH_2_), 54.00 (piperazine CH_2_), 54.18 (piperazine CH_2_), 54.47 (piperazine CH_2_), 56.72 (OMe), 57.92 (C-3′′), 59.49 (C-1′′′′), 91.78 (C-7′), 99.02 (C-3′′′′), 105.15 (C-5′), 118.14 (C-4′′′′′a), 122.71 (C-3′), 123.48 (C-5′′′′′), 125.05 (C-6′′′′′), 125.34 (C-3), 127.57 (C-6), 128.18 (C-5), 128.65 (C-8′′′′′), 128.90 (C-8), 129.15 (C-4′a), 129.47 (C-4a), 132.87 (C-4), 134.65 (C-8′a), 135.04 (C-7′′′′′), 135.30 (C-7), 144.64 (C-4), 144.97 (C-2′), 148.15 (C-8′), 149.84 (C-8a), 151.35 (C-4′′′′′), 151.90 (C-2), 152.72 (C-2′′′′′), 156.85 (C-6′) ppm. MS (EI, 70 eV): m/z (%) = 682.1/681.1/680.1/679.1 [M]^+•^ (10/20/15/28), 489.1/488.1 [M-C_10_H_8_ClN_2_]^+^ (10/20), 364.0/363.0/362.0 [C_21_H_17_ClN_3_O]^+^ (15/15/42), 350.0/349.0/348.0/347.0 [C_20_H_15_ClN_3_O]^+^ (13/17/29/11), 337.0/336.0/335.0 [C_19_H_14_ClN_3_O]^+•^ (13/20/27), 250.1/249.1/248.1 [C_13_H_15_ClN_3_]^+^ (35/18/100), 193.0/192.0/191.0 [C_10_H_8_ClN_2_]^+^ (17/60/28), HRMS (ESI) calcd. [M+H]^+^ 680.26659; found 680.26636.

#### 3.2.25. (4-(Bromomethyl)phenyl)methanol (**31**)

PPh_3_ (7.557 g, 0.029 mol) was added to a solution of 1,4-benzenedimethanol (9.907 g, 0.072 mol) and (CBrCl_2_)_2_ (7.770 g, 0.024 mol) in DCM (dry, 120 mL), and the reaction mixture was stirred at 25°C overnight. The solvent was concentrated and the residue purified with flash column chromatography on silica gel (petroleum ether/ethyl acetate 10 : 1) to obtain compound** 31** (3.020 g, 0.015 mol, 63%) as a colorless solid. Mp 79°C (petroleum ether/ethyl acetate), IR (ATR-FTIR): $\tilde{v}=3313$ (m, br), 2921 (w), 2859 (w), 2360 (w), 2337 (w), 1918 (w), 1683 (w), 1513 (w), 1442 (w), 1417 (m), 1349 (m), 1301 (w), 1222 (m), 1193 (m), 1093 (w), 1000 (s), 871 (w), 829 (s), 757 (m), 727 (s) cm^−1^. ^1^H-NMR (400 MHz, CDCl_3_): *δ* = 1.69 (s, 1 H, OH), 4.48 (s, 2 H, CH_2_Br), 4.67 (s, 2 H, CH_2_OH), 7.32 (d, ^3^ *J*_H–H_ = 8.24 Hz, 2 H, Ph-H), 7.37 (d, ^3^ *J*_H–H_ = 8.20 Hz, 2 H, Ph-H) ppm. ^13^C-NMR (100 MHz, CDCl_3_): *δ* = 33.43 (CH_2_Br), 65.11 (CH_2_OH), 127.53 (Ph-CH), 129.47 (Ph-CH), 137.40 (C-4), 141.40 (C-1) ppm. MS (EI, 70 eV): m/z (%) = 202.0/200.0 [M]^+•^ (3/3), 122.0/121.0 [M-Br]^+^ (10/100), 92.0/91.0 [C_7_H_7_]^+^ (6/36), HRMS (EI) calcd. [M]^+•^ 199.98313; found 199.98343.

#### 3.2.26. 1,4-Bis(bromomethyl)benzene (**32**)

Within the synthesis of compound** 31**, the dibrominated side product** 32** was given in 35% yield (2.229 g, 0.008 mmol). Mp 144°C (petroleum ether/ethyl acetate), IR (ATR-FTIR): $\tilde{v}=2971$ (w), 2360 (w), 1976 (w), 1924 (w), 1806 (w), 1691 (w), 1511 (w), 1434 (w), 1419 (w), 1261 (w), 1224 (w), 1195 (w), 1126 (w), 1083 (w), 1020 (w), 968 (w), 846 (m), 794 (w), 750 (s), 667 (w), 605 (m) cm^−1^. ^1^H-NMR (400 MHz, CDCl_3_): *δ* = 4.46 (s, 4H, CH_2_), 7.35 (s, 4 H, Ph-H) ppm. ^13^C-NMR (100 MHz, CDCl_3_): *δ* = 32.98 (CH_2_), 129.70 (Ph-CH), 138.24 (C-1, C-4) ppm. MS (EI, 70 eV): m/z (%) = 265.9/263.9/261.9 [M]^+•^ (3/6/3), 186.0/185.0/184.0/183.0 [M-Br]^+^ (6/71/8/74), 105.1/104.1 [C_8_H_8_]^+•^ (9/100), HRMS (EI) calcd. [M]^+^ 261.89873; found 261.89852.

#### 3.2.27. (4-(Azidomethyl)phenyl)methanol (**33**)

NaN_3_ (411.1 mg, 6.34 mmol) was added to a solution of the brominated compound** 31** (424.7 mg, 2.11 mmol) in DMF (dry, 7 mL) and the reaction mixture was stirred at 25°C overnight. The complete conversion was determined by ^1^H-NMR spectroscopy. The solvent was azeotropically removed using toluene under reduced pressure, and the residue was suspended in dichloromethane, filtered (Celite), and concentrated. Purification with flash column chromatography on silica gel (ethyl acetate 100%) gave the colorless solid** 33** (341.7 mg (2.09 mmol, 99%). Mp 76°C (ethyl acetate), IR (ATR-FTIR): $\tilde{v}=3332$ (w, br), 3023 (w), 2929 (w), 2873 (w), 2092 (s), 1616 (w), 1513 (w), 1448 (w), 1419 (w), 1344 (w), 1255 (m), 1207 (m), 1110 (w), 1010 (m), 877 (w), 804 (m), 752 (s), 657 (m) cm^−1^. ^1^H-NMR (400 MHz, CDCl_3_): *δ* = 1.78 (s, 1 H, OH), 4.31 (s, 2 H, CH_2_N_3_), 4.68 (s, 2 H, CH_2_OH), 7.29 (d, ^3^ *J*_H–H_ = 8.12 Hz, 2 H, Ph-H), 7.36 (d, ^3^ *J*_H–H_ = 8.12 Hz, 2 H, Ph-H) ppm. ^13^C-NMR (100 MHz, CDCl_3_): *δ* = 54.73 (CH_2_N_3_), 65.11 (CH_2_OH), 127.58 (Ph-CH), 128.67 (Ph-CH), 134.91 (C-4), 141.25 (C-1) ppm. MS (EI, 70 eV): m/z (%) = 163.0 [M]^+•^ (7), 122.0/121.0 [M-N_3_]^+^ (3/31), 18.0 [H_2_O]^+•^ (100), HRMS (EI) calcd. [M]^+•^ 163.07401; found 163.07374.

#### 3.2.28. 4-(Azidomethyl)benzyl 4-Methylbenzenesulfonate (**34**)

NaH (0.823 g of a 55% oily dispersion, 0.018 mol) was added in portions to a solution of azide** 33** (1.014 g, 0.006 mol) and 4-toluenesulfonyl chloride (1.421 g, 0.007 mol) in DCM (dry, 20 mL) at 0°C under nitrogen atmosphere. The reaction mixture was allowed to warm up to room temperature and stirred for further 2.5 hours. The excess of NaH was reacted with glacial acetic acid at 0°C until no more gas was generated; the mixture was filtered (Celite) and concentrated. Purification of the residue with flash column chromatography on silica gel (petroleum ether/ethyl acetate 10 : 1) gave compound** 34** (1.352 g, 71%) as colorless oil. IR (ATR-FTIR): $\tilde{v}$ = 3060–2920 (w, br), 2360 (w), 2341 (w), 2096 (m), 1914 (w), 1798 (w), 1732 (w), 1699 (w), 1644 (w), 1596 (w), 1516 (w), 1492 (w), 1477 (w), 1462 (w), 1449 (w), 1426 (w), 1397 (w), 1350 (s), 1325 (w), 1301 (w), 1290 (w), 1247 (m), 1215 (w), 1184 (m), 1170 (s), 1116 (w), 1094 (m), 1039 (w), 1018 (w), 954 (w), 928 (s), 885 (w), 862 (s), 848 (m), 820 (m), 808 (m), 788 (m), 762 (s), 732 (m), 702 (w), 664 (s), 639 (w) cm^−1^. ^1^H-NMR (400 MHz, CD_2_Cl_2_): *δ* = 2.45 (s, 3 H, Me), 4.34 (s, 2 H, CH_2_N_3_), 5.05 (s, 2 H, CH_2_OTs), 7.26–7.31 (m, 4 H, Ph-H), 7.36 (d, ^3^ *J*_H–H_ = 7.96 Hz, 2 H, Ph-H), 7.78 (d, ^3^ *J*_H–H_ = 8.32 Hz, 2 H, Ph-H) ppm. ^13^C-NMR (100 MHz, CD_2_Cl_2_): *δ* = 21.93 (Me), 54.86 (CH_2_N_3_), 72.09 (CH_2_OTs), 128.42 (Ph-CH), 128.99 (Ph-CH), 129.47 (Ph-CH), 130.48 (Ph-CH), 133.74 (Ph-C), 134.16 (Ph-C), 137.12 (Ph-C), 145.75 (Ph-C) ppm. MS (EI, 70 eV): m/z (%) = 276.2/275.2 [M-N_3_]^+^ (6/32), 163.1/162.1 [M-Ts]^+^ (4/33), 146.1 [C_8_H_8_N_3_]^+^ (26), 132.1 [C_7_H_6_N_3_]^+^ (10), 120.1/119.1 [C_8_H_7_O]^+^ (6/63), 105.0/104.1 [C_8_H_8_]^+^ (3/13), 92.0/91.1 [C_7_H_7_]^+^ (7/100), 78.1/77.1 [C_6_H_5_]^+^ (5/13), HRMS (EI) calcd. [M]^+^ 275.07364; found 275.07334.

#### 3.2.29. (4′-((6′′′-Methoxyquinolin-8′′′-ylamino)methyl)phenyl)methanol (**36**)

A mixture consisting of 6-methoxy-8-aminoquinoline (**4**, 200.3 mg, 1.15 mmol), the brominated alcohol** 31** (254.8 mg, 1.27 mmol), and NaOAc (282.2 mg, 3.44 mmol) in DMF (dry, 5 mL) was stirred at 25°C for 17 hours. The solvent DMF was removed azeotropically with toluene under reduced pressure; the residue was suspended in dichloromethane, filtered (Celite), and concentrated. Purification with flash column chromatography on deactivated silica gel (petroleum ether/ethyl acetate 4 : 1) gave compound** 36** (229.3 mg, 68%) as yellow oil. IR (ATR-FTIR): $\tilde{v}=3394$ (w, br), 3050 (w), 3005 (w), 2930 (w, br), 2857 (w, br), 2513 (w, br), 1725 (w), 1612 (s), 1576 (s), 1516 (m), 1500 (s), 1456 (m), 1419 (m), 1405 (m), 1385 (s), 1338 (w), 1298 (w), 1262 (w), 1238 (w), 1207 (s), 1155 (m), 1126 (w), 1056 (m), 1029 (m), 1014 (m), 966 (w), 922 (w), 901 (w), 818 (s), 787 (s), 747 (m), 698 (w), 667 (w), 624 (w) cm^−1^. ^1^H-NMR (400 MHz, MeOD-d_4_): *δ* = 3.80 (s, 3 H, OMe), 4.49 (s, 2 H, 1′′-CH_2_), 4.57 (s, 2 H, 1-CH_2_), 6.22 (d, ^4^ *J*_H–H_ = 2.04 Hz, 1 H, 7′′′-H), 6.45 (d, ^4^ *J*_H–H_ = 2.46 Hz, 1 H, 5′′′-H), 7.31 (d, ^3^ *J*_H–H_ = 8.04 Hz, 2 H, 2′-H, 6′-H), 7.35 (dd, ^3^ *J*_H–H_ = 4.20 Hz, 8.28 Hz, 1 H, 3′′′-H), 7.39 (d, ^3^ *J*_H–H_ = 8.10 Hz, 2 H, 3′-H, 5′-H), 8.01 (dd, ^3^ *J*_H–H_ = 8.28 Hz, ^4^ *J*_H–H_ = 1.44 Hz, 1 H, 4′′′-H), 8.51 (dd, ^3^ *J*_H–H_ = 4.14 Hz, ^4^ *J*_H–H_ = 1.38 Hz, 1 H, 2′′′-H) ppm. ^13^C-NMR (100 MHz, MeOD-d_4_): *δ* = 48.20 (C-1′′), 55.73 (OMe), 65.21 (C-1), 93.77 (C-5′′′), 99.14 (C-7′′′), 123.08 (C-3′′′), 128.50 (C-3′, C-5′), 128.54 (C-2′, C-6′), 131.52 (C-4′a), 136.39 (C-4′), 136.66 (C-8′a), 139.92 (C-4′), 141.66 (C-1′), 145.70 (C-2′′′), 146.86 (C-8′′′), 160.93 (C-6′′′) ppm. MS (EI, 70 eV): m/z (%) = 297.2/296.2/295.2/294.2 [M]^+•^ (18/52/100/87), 265.1/264.1/263.1 [M-CH_3_O]^+^ (7/11/8), 189.1/188.1/187.1 [C_11_H_11_N_2_O]^+^ (6/18/22), 175.1/174.1 [C_10_H_10_N_2_O]^+•^ (5/5), 160.1/159.1 [C_9_H_7_N_2_O]^+•^ (54/55), 92.1/91.1 [C_7_H_7_]^+^ (8/20), 87.1/77.1 [C_6_H_5_]^+^ (9/19), HRMS (ESI) calcd. [M+H]^+^ 295.14410; found 295.14401.

#### 3.2.30. *N*-(4′-(Azidomethyl)benzyl)-6-methoxyquinolin-8-amine (**35**)

Compound** 35** was obtained by two synthetic routes that are described in the following procedures.

*Method A*. The tosylated azide** 34** (905.3 mg, 2.85 mmol) was added to a suspension of 6-methoxy-8-aminoquinoline (**4**, 454.7 mg, 2.61 mmol) and NaOAc (641.3 mg, 7.82 mmol) in DMF (dry, 10 mL), and the reaction mixture was stirred at 25°C for 12 hours. The solvent was azeotropically removed with toluene under reduced pressure; the residue was suspended in dichloromethane, filtered (Celite), and concentrated. Purification with flash column chromatography on silica gel (petroleum ether/ethyl acetate) gave compound** 35** (343.4 mg, 41%) as yellow oil.

*Method B*. Diphenylphosphoryl azide (DPPA, 33.1 *μ*L, 42.3 mg, 0.15 mmol) and 1,8-diazabicyclo[5.4.0]undec-7-en (DBU, 23.0 *μ*L, 23.4 mg, 0.15 mmol) were added to a solution of alcohol** 36** (37.7 mg, 0.13 mmol) in toluene (dry, 5 mL), and the mixture was stirred at 25°C for 3.5 hours. The solvent was concentrated and the residue purified using flash column chromatography on silica gel (petroleum ether/ethyl acetate 10 : 1) to obtain compound** 35** (32.2 mg, 0.10 mmol, 78%) as yellow oil. IR (ATR-FTIR): $\tilde{v}=3395$ (w, br), 3049–2852 (w, br), 2093 (s), 1616 (m), 1593 (m), 1576 (m), 1517 (s), 1457 (m), 1420 (m), 1386 (m), 1336 (w), 1286 (w), 1254 (w), 1238 (w), 1209 (s), 1163 (m), 1153 (m), 1124 (w), 1060 (w), 1022 (w), 999 (w), 967 (w), 935 (w), 902 (w), 880 (w), 820 (m), 789 (m), 667 (w), 644 (w), 624 (w), 603 (w) cm^−1^. ^1^H-NMR (600 MHz, CD_2_Cl_2_): *δ* = 3.83 (s, 3 H, OMe), 4.34 (s, 2 H, CH_2_N_3_), 4.54 (d, ^3^ *J*_H–H_ = 5.94 Hz, 2 H, CH_2_NHR), 6.22 (d, ^3^ *J*_H–H_ = 2.52 Hz, 1 H, 7-H), 6.40 (d, ^3^ *J*_H–H_ = 2.52 Hz, 1 H, 5-H), 6.65–6.68 (m, 1 H, NH), 7.31 (d, ^3^ *J*_H–H_ = 8.10 Hz, 2 H, 3′-H, 5′-H), 7.34 (d, ^3^ *J*_H–H_ = 4.20 Hz, 8.22 Hz, 1 H, 3-H), 7.44 (d, ^3^ *J*_H–H_ = 8.04 Hz, 2 H, 2′-H, 6′-H), 7.97 (dd, ^3^ *J*_H–H_ = 8.28 Hz, ^4^ *J*_H–H_ = 1.62 Hz, 1 H, 4-H), 8.55 (dd, ^3^ *J*_H–H_ = 4.20 Hz, ^4^ *J*_H–H_ = 1.62 Hz, 1 H, 2-H) ppm. ^13^C-NMR (150 MHz, CD_2_Cl_2_): *δ* = 47.57 (CH_2_NHR), 55.02 (CH_2_N_3_), 55.66 (OMe), 92.87 (C-5), 97.70 (C-7), 122.51 (C-3), 128.17 (C-2′, C-6′), 129.05 (C-3′, C-5′), 130.26 (C-4a), 134.98 (C-4′), 135.21 (C-4), 135.78 (C-8a), 139.98 (C-1′), 145.11 (C-2), 145.98 (C-8), 159.81 (C-6) ppm. MS (EI, 70 eV): m/z (%) = 320.1/319.1 [M]^+•^ (21/100), 278.1/277.1 [M-N_3_]^+^ (4/17), 188.1/187.1 [C_11_H_11_N_2_O]^+^ (3/25), 160.1/159.1 [C_10_H_9_NO]^+•^ (15/65), 91.1 [C_7_H_7_]^+^ (24), 78.1/77.1 [C_6_H_5_]^+^ (4/5), HRMS (ESI) calcd. [M+H]^+^ 320.15059; found 320.15042.

#### 3.2.31. *N*-(4′-(Aminomethyl)benzyl)-6-methoxyquinolin-8-amine (**37**)

PPh_3_ (156.9 mg, 0.60 mmol) was added to a solution of azide** 35** (158.9 mg, 0.50 mmol) in MeOH (dry, 5 mL) and the mixture was stirred at 25°C for 2 hours. The solvent was removed under reduced pressure, and the residue was purified on deactivated silica gel (ethyl acetate 100%) to give compound** 37** (140.1 mg, 95%) as yellow oil. IR (ATR-FTIR): $\tilde{v}=3383$ (w), 3310 (w), 3004–2853 (w, br), 2360 (w), 2342 (w), 1617 (m), 1576 (m), 1518 (s), 1461 (m), 1420 (m), 1387 (m), 1350 (w), 1293 (w), 1276 (w), 1261 (w), 1237 (w), 1210 (s), 1163 (s), 1122 (w), 1059 (w), 1022 (w), 1000 (w), 936 (w), 893 (w), 821 (m), 789 (m), 669 (w), 627 (w), 614 (w), 604 (w) cm^−1^. ^1^H-NMR (600 MHz, CD_2_Cl_2_): *δ* = 1.46 (s, br, 2 H, NH_2_), 3.82-3.83 (m, 5 H, OMe, CH_2_NH_2_), 4.50 (d, ^3^ *J*_H–H_ = 5.94 Hz, 2 H, CH_2_NH), 6.23 (d, ^4^ *J*_H–H_ = 2.46 Hz, 1 H, 7-H), 6.39 (d, ^4^ *J*_H–H_ = 2.52 Hz, 1 H, 5-H), 6.62 (s, br, 1 H, NH), 7.29 (d, ^3^ *J*_H–H_ = 7.98 Hz, 2 H, 3′-H, 5′-H), 7.33 (dd, ^3^ *J*_H–H_ = 4.14 Hz, 8.22 Hz, 1 H, 3-H), 7.37 (d, ^3^ *J*_H–H_ = 8.04 Hz, 2 H, 2′-H, 6′-H), 7.96 (dd, ^3^ *J*_H–H_ = 8.28 Hz, ^4^ *J*_H–H_ = 1.56 Hz, 1 H, 4-H), 8.53 (dd, ^3^ *J*_H–H_ = 4.20 Hz, ^4^ *J*_H–H_ = 1.62 Hz, 1 H, 2-H) ppm. ^13^C-NMR (150 MHz, CD_2_Cl_2_): *δ* = 46.63 (CH_2_NH_2_), 47.64 (CH_2_NH), 55.65 (OMe), 92.72 (C-5), 97.63 (C-7), 122.47 (C-3), 127.82 (C-3′, C-5′), 127.90 (C-2′, C-6′), 130.25 (C-4a), 135.19 (C-4), 135.80 (C-8a), 138.00 (C-1′), 143.36 (C-4′), 145.06 (C-2), 146.07 (C-8), 159.83 (C-6) ppm. MS (EI, 70 eV): m/z (%) = 295.1/294.1/293.1/292.1 [M]^+•^ (3/25/100/32), 187.0 [M-C_7_H_8_N]^+^ (17), 160.0/159.0 [C_10_H_9_NO]^+•^ (16/43), 120.1 [C_8_H_10_N]^+^ (14), 91.1 [C_7_H_7_]^+^ (14), HRMS (ESI) calcd. [M+H]^+^ 294.16009; found 294.15997.

#### 3.2.32. 7-Chloro-*N*-(4′′-((6′′-methoxyquinolin-8′′-ylamino)methyl)benzyl)quinolin-4-amine (**30**)

Amine** 37** (80.6 mg, 0.27 mmol) and 4,7-dichloroquinoline (41.9 mg, 0.21 mmol) were stirred at 120°C under neat conditions for 13.5 hours. The reaction mixture was suspended in dichloromethane and methanol, alkalized using ammonia solution and all volatile components were removed under reduced pressure. The residue was repeatedly suspended in dichloromethane and methanol, filtered (Celite), and concentrated. Purification with flash column chromatography on deactivated silica gel (gradient elution with petroleum ether/ethyl acetate 5 : 1, followed by petroleum ether/ethyl acetate 2 : 1) gave hybrid compound** 30** (41.1 mg, 43%) as beige crystals. Mp 273°C (petroleum ether/ethyl acetate), IR (ATR-FTIR): $\tilde{v}=2926$ (w, br), 2850 (w, br), 2528 (w), 2358 (w, br), 2340 (w, br), 1734 (w), 1608 (m), 1574 (s), 1509 (m), 1467 (m), 1453 (m), 1419 (m), 1388 (m), 1363 (m), 1333 (m), 1283 (m), 1206 (m), 1154 (m), 1128 (w), 1061 (m), 1018 (m), 956 (w), 930 (w), 896 (m), 866 (w), 845 (w), 815 (m), 806 (s), 788 (m), 761 (w), 732 (w), 671 (w), 644 (w), 634 (w), 623 (w) cm^−1^. ^1^H-NMR (600 MHz, CD_2_Cl_2_/MeOD-d_4_ = 10 : 1): *δ* = 3.81 (s, 3 H, OMe), 4.50 (s, 2 H, CH_2_NH-PQ), 4.54 (s, 2 H, CH_2_NH-CQ), 6.22 (d, ^4^ *J*_H–H_ = 2.04 Hz, 1 H, 7′′-H), 6.39–6.42 (m, 2 H, 3-H, 5′′-H), 7.33–7.36 (m, 3 H, 2′-H, 6′-H, 3′′-H), 7.38–7.41 (m, 3 H, 6-H, 3′-H, 5′-H), 7.86 (d, ^4^ *J*_H–H_ = 2.04 Hz, 1 H, 8-H), 7.91 (d, ^3^ *J*_H–H_ = 8.94 Hz, 1 H, 5-H), 7.97 (dd, ^3^ *J*_H–H_ = 8.28 Hz, ^4^ *J*_H–H_ = 1.32 Hz, 1 H, 4′′-H), 8.33 (d, ^3^ *J*_H–H_ = 5.52 Hz, 1 H, 2-H), 8.51 (dd, ^3^ *J*_H–H_ = 4.26 Hz, ^4^ *J*_H–H_ = 1.56 Hz, 1 H, 2′′-H) ppm. ^13^C-NMR (150 MHz, CD_2_Cl_2_/MeOD-d_4_ = 10 : 1): *δ* = 47.31 (CH_2_-NH-CQ), 47.51 (CH_2_-NH-PQ), 55.67 (OMe), 93.03 (C-5′′), 97.98 (C-7′′), 99.94 (C-3), 117.74 (C-4a), 122.54 (C-3′′), 122.69 (C-5), 125.97 (C-6), 127.59 (C-8), 128.13 (C-2′, C-6′), 128.28 (C-3′, C-5′), 130.39 (C-4′′a), 135.51 (C-4′′), 135.69 (C-8′′a), 135.88 (C-7), 136.89 (C-1′), 139.29 (C-4′), 145.16 (C-2′′), 145.91 (C-8′′), 148.55 (C-8a), 151.25 (C-4), 151.56 (C-2), 159.79 (C-6′′) ppm. MS (EI, 70 eV): m/z (%) = 455.9/454.9/453.9 [C_27_H_23_ClN_4_O]^+•^ (2/3/5), 279.0/278.0/277.0/276.0 [C_18_H_17_N_2_O]^+^ (1/7/36/100), 160.0/159.0 [C_10_H_9_NO]^+•^ (2/7), HRMS (ESI) calcd. [M+H]^+^ 455.16332; found 455.16320.

#### 3.2.33. 7-Chloroquinolin-4-amine (**3**) [75]

Gaseous ammonia was directed through a solution of 4,7-dichloroquinoline (11.907 g, 0.060 mmol) in phenol (58.000 g, 0.616 mol) at 170°C; the mixture was heated up to 200°C and stirred for 2.5 hours. After addition of glacial acetic acid (15 mL), water (30 mL), and diethyl ether (100 mL), a colorless solid was obtained and filtered. The residue was dissolved in water, alkalized using aqueous NaOH solution, and exhaustively extracted using diethyl ether. The combined organic extracts were dried (MgSO_4_) and concentrated. Recrystallization (H_2_O) gave** 3** (8.359 g, 78%) as colorless crystals. Mp 152°C (H_2_O), IR (ATR-FTIR): $\tilde{v}=3447$ (w), 3355 (w), 3060 (w, br), 1637 (m), 1612 (m), 1574 (s), 1507 (m), 1442 (m), 1369 (w), 1326 (m), 1284 (m), 1200 (m), 1129 (w), 1077 (w), 1019 (w), 909 (m), 877 (m), 855 (m), 837 (m), 812 (s), 760 (m), 732 (m), 643 (m), 626 (m) cm^−1^. ^1^H-NMR (400 MHz, CDCl_3_): *δ* = 6.56 (d, ^3^ *J*_H–H_ = 5.16 Hz, 1 H, 3-H), 7.37 (dd, ^3^ *J*_H–H_ = 8.96 Hz, ^4^ *J*_H–H_ = 2.16 Hz, 1 H, 6-H), 7.67 (d, ^3^ *J*_H–H_ = 8.96 Hz, 1 H, 5-H), 7.96 (d, ^4^ *J*_H–H_ = 2.04 Hz, 1 H, 8-H), 8.49 (d, ^3^ *J*_H–H_ = 5.20 Hz, 1 H, 2-H) ppm. ^13^C-NMR (100 MHz, CDCl_3_): *δ* = 104.18 (C-3), 117.34, 121.89, 125.91, 128.99, 135.49 (C-7), 149.71, 149.84 (C-4), 151.96 (C-2) ppm. ^15^N-NMR: (40.5 MHz, DMSO-d_6_): −310.0 (4-NH_2_), −110.0 (N-1) ppm. MS (EI, 70 eV): m/z (%) = 180.1/179.1/178.1 [M]^+•^ (34/14/100).
